# Potentiating doxorubicin activity through BCL-2 inhibition in p53 wild-type and mutated triple-negative breast cancer

**DOI:** 10.3389/fonc.2025.1549282

**Published:** 2025-04-02

**Authors:** Anna R. Schreiber, Stephen G. Smoots, Marilyn M. Jackson, Stacey M. Bagby, Evan D. Dus, Adrian T. A. Dominguez, Cameron A. Binns, Todd M. Pitts, Jennifer R. Diamond

**Affiliations:** Division of Medical Oncology, University of Colorado, Aurora, CO, United States

**Keywords:** senescence, doxorubicin, TNBC, venetoclax, resistance

## Abstract

**Background:**

Triple-negative breast cancer (TNBC) is an aggressive sub-type of breast cancer that is associated with higher rates of recurrent disease. Chemotherapy with an anthracycline is an integral part of curative therapy but resistance remains a clinical problem. Cellular senescence is a terminal cell fate that has been observed in models of doxorubicin resistance. Identifying novel combinations with doxorubicin to eliminate senescent cells and promote apoptosis may lead to improved clinical outcomes. The purpose of this study was to investigate the combination of doxorubicin with the pro-apoptotic BCL-2 inhibitor venetoclax in TNBC cell lines and to assess the role of p53 in cellular senescence and apoptosis.

**Methods:**

TNBC cell lines with wild-type (WT), mutated or knocked-down (KD) p53 were treated with doxorubicin, venetoclax or the combination *in vitro* and evaluated for impacts on viability, proliferation, apoptosis, and senescence. Down-stream markers of apoptosis were also assessed to evaluate cellular mechanistic changes. An *in vivo* TNBC MDA-MB-231 murine model was used to assess tumor growth, cellular proliferation, and senescence changes following treatment with doxorubicin, venetoclax or combination.

**Results:**

Venetoclax with doxorubicin had synergistic antiproliferative activity against TNBC cell lines and increased apoptosis. The addition of venetoclax to doxorubicin reduced senescent cells in a p53-independent manner. *In vivo*, the addition of venetoclax to doxorubicin improved tumor growth inhibition and reduced senescent cells.

**Conclusion:**

The combination of doxorubicin with venetoclax is promising for the treatment of p53-WT and mutated TNBC and this work supports further investigation.

## 1 Introduction

Triple-negative breast cancer (TNBC) is an aggressive sub-type of breast cancer that lacks the expression of the hormone receptors (HR) estrogen and progesterone, as well as over-expression of human epidermal growth factor receptor 2 (HER2). Compared to other breast cancer sub-types, TNBC is associated with worse overall survival (OS) as well as higher rates of disease recurrence (1, 2). Until recently, treatment options for TNBC were limited to only cytotoxic chemotherapies. Immunotherapy has emerged as a promising treatment modality and is now widely used in high-risk TNBC and metastatic TNBC (3–5). In addition, antibody-drug conjugates (ADCs) and poly (ADP-ribose) polymerase (PARP) inhibitors have also improved survival in metastatic TNBC (6–9). Despite these advances, chemotherapy with an anthracycline remains an integral part of curative therapy and reducing risk of recurrence (10). However, even with the use of anthracyclines and taxanes, patients with TNBC can have early relapse and develop resistance leading to disease progression (11, 12). Resistance to chemotherapy, including anthracyclines, is a major barrier to effective treatment and methods to overcome this resistance need to be developed.

Cellular senescence is a phenomenon where cells remain in an active and arrested state. It is well established that treatment with doxorubicin can result in therapy induced cellular senescence in TNBC (12–15). Studies have demonstrated that sub-populations of these arrested, senescent cells, do not remain in a state of permanent cycle arrest and can re-activate, leading to tumor progression and disease recurrence (16, 17). In addition, these arrested, senescent cells can secrete pro-inflammatory markers known as the senescence-associated secretory phenotype (SASP) that can promote an environment ideal for tumor growth and relapse (13, 18). From these observations, it can be concluded that doxorubicin induced cellular senescence is a potential mechanism of cellular resistance and progression. In TNBC, the upregulation of HIST1H2BK (histone cluster 1 H2B family member k), ubiquitin specific protease (USP) 7, and cellular FLICE-like inhibitory protein (cFLIP) have all been shown to contribute to doxorubicin resistance (19–21). Combination strategies using HDAC inhibitors and USP7 inhibitors have shown synergistic effects in preclinical experiments (19, 20, 22). While other mechanisms of resistance have been studied, doxorubicin resistance remains a clinical issue for patients. Understanding how to convert cells from a state of senescence to a state of apoptosis could be useful for overcoming doxorubicin-mediated resistance in TNBC patients and needs to be further investigated.

Senolytic agents are a class of drugs that can induce apoptosis of senescent cells. Venetoclax is an inhibitor of the anti-apoptotic protein, BCL-2, and is a known pro-apoptotic and senolytic agent (23, 24). In binding to BCL-2, venetoclax displaces pro-apoptotic proteins and triggers the activation of cell death (25). Currently venetoclax has FDA approval for the treatment of hematologic malignancies such as chronic lymphocytic leukemia (CLL) and acute myeloid leukemia (AML) (26). BCL-2 is over-expressed in TNBC, making it an attractive target (27). BCL-2 inhibitors have been explored in TNBC cell lines with other targeted agents such as gamma-secretase inhibitors, WEE-1 inhibitors, and neratinib and have shown synergistic effects (28–31).

Mutations in *TP53* occur in approximately 80% of TNBC (32, 33). Following DNA damage, p53 is activated and induces apoptosis in normal cells. Activated p53 has been found to play a role in modulating senescence by activating RB via p21 which in turn halts transcription of E2F resulting in senescence (34, 35). Studies have demonstrated that cells wild-type (WT) for p53 enter senescence following treatment with doxorubicin, therefore escaping apoptosis (36, 37). It remains unclear how mutated p53 may influence cellular senescence.

Initial efforts focused on limited TNBC cell lines demonstrate that senolytic agents can potentiate the activity of doxorubicin in TNBC (17, 37–39). The purpose of this study was to evaluate the combination of doxorubicin with the BCL-2 inhibitor venetoclax in an extended panel of p53 WT and mutated TNBC cell lines to investigate the role of p53 in mediating apoptotic and senescent responses.

## 2 Materials and methods

### 2.1 Cell lines

Human TNBC cell lines used for analysis included MDA-MB-436 (RRID: CVCL_0623), MDA-MB-231 (RRID: CVCL_0062), MDA-MB-157 (RRID: CVCL_0618), HCC1395 (RRID: CVCL_1249), HCC1937 (RRID: CVCL_0290), HCC38 (RRID: CVCL_1267), HCC1187 (RRID: CVCL_1247), HCC1143 (RRID: CVCL_1245), HCC1395 (RRID: CVCL_1249), Hs 578T (RRID: CVCL_0332), BT549 (RRID: CVCL_1092) (obtained from American Type Culture Collection, Manassas, VA, USA), CAL-85–1 (RRID: CVCL_1114), CAL-51 (RRID: CVCL_1110), CAL-148 (RRID: CVCL_1106), CAL-120 (RRID: CVCL_1104), HDQ-P1 (RRID: CVCL_2067) (obtained from the Deutsche Sammlung von Mikroorganismen und Zellkulturen GmbH, Braunschweig, Germany), BT20 (RRID: CVCL_0178), HCC70 (RRID: CVCL_1270), and MDA-MB-468 (RRID: CVCL_0419) (obtained from University of Colorado Cancer Center Cell Technologies Core, Aurora, Colorado, USA). The CAL-51 P53-10 shRNA knock-down (KD) model and CAL-51 scramble (SCR) were made as previously described (22, 40). Cells were cultured in Corning Dulbecco’s Modified Eagle Media (DMEM) containing 10% fetal bovine serum (FBS) (Atlas Biologicals), 1% Normocin (InvivoGen), 1% penicillin/streptomycin (Gibco), and 1% nonessential amino acids (Corning). Cell lines were tested for mycoplasma regularly while continuously maintained in a 37°C incubator with 5% CO_2_. Cells were not passed beyond passage 20.

### 2.2 Drugs

For *in vitro* studies, both venetoclax (#S8048) and doxorubicin (#S1208) were purchased from Selleck Chemicals (Houston, TX, USA), and dissolved in DMSO to a stock concentration of 10 mM. For *in vivo* studies, venetoclax was purchased from Selleck Chemicals and was sonicated in 1ml (5%) DMSO until fully dissolved, followed by 10ml PEG300 (50%), 1ml Tween 80 (5%), and 8ml (40%) HPLC water. The final drug mixture was then sonicated at room temperature for one hour and stirred overnight at 4°C. Doxorubicin was purchased from the University of Colorado Research Pharmacy and diluted 1:5.8 in sterile saline (Aurora, CO).

### 2.3 Viability assays and synergy

To determine cell viability, selected TNBC cell lines were seeded at 8,000 cells per well in a white-walled 96-well plate. Cells were immediately dosed at varying concentrations of doxorubicin (0.03125 µM, 0.0625 µM, 0.125 µM, 0.25 µM, 0.5 µM) and venetoclax (12.5 µM, 25 µM, 50 µM). After 72 hours, cell viability was assessed using BioTek Synergy H1 plate reader and CellTiter-Glo Luminescent Cell Viability Assay from Promega (Madison, WI, USA). 72 hours was selected as the time point as this roughly allows for three doubling times. Synergy Finder+ (https://www.synergyfinderplus.org/) was used to calculate the average synergy score across experiments. All experiments were repeated three times. A score > 10 indicates synergy while a score < -10 is antagonistic.

### 2.4 Proliferation assays

Selected TNBC cell lines were seeded at 4,000 cells per well in a white-walled 96-well plate. 24 hours after plating, cells were dosed with no drug (ND), 0.5 µM of doxorubicin, 25 µM of venetoclax, or 0.5 µM of doxorubicin in combination with 25 µM of venetoclax. Plates were placed on the Agilent BioTek Biospa live cell analysis system and proliferation for live cells was assessed using Agilent BioTek’s high contrast cell counting every 4 hours for a total of 48 hours. Each experiment was replicated three times.

### 2.5 Annexin analysis

150,000 cells were plated in 6-well plates and after 24 hours were dosed with ND, 0.5 µM of doxorubicin, 25 µM of venetoclax, or 0.5 µM of doxorubicin in combination with 25 µM of venetoclax. Cells were treated with drug for 24 and 48 hours and then adherent cells and cell pellets from centrifuged media were collected and washed with PBS. Cells were then resuspended with Annexin-V-FITC and PI (Cell Signaling Technology, Danvers, MA #6592). CAL-51 SCR and CAL-51 p53-10 KD cell lines were resuspended in Annexin-V-APC and PI (Biolegend, San Diego, CA #640,919) given the presence of GFP in the SCR and KD constructs. All cell lines were then analyzed using flow cytometry (UCCC Flow Cytometry Shared Resource) and each experiment was replicated three times.

### 2.6 Cell cycle analysis

150,000 cells were plated on a 6-well plate and given 24 hours to adhere. Cells were treated with ND, 0.5 µM of doxorubicin, 25 µM of venetoclax, or 0.5 µM of doxorubicin in combination with 25 µM of venetoclax for 24 hours. Cells were then collected, washed with PBS, and fixed with 70% methanol. Cells were maintained on ice for 15 minutes and then 50 µg/mL of RNase (ThermoFisher Scientific, Waltham, MA) and 1mg/mL of PI (Invitrogen, Waltham, MA) was added. Cells were left to rest at room temperature for 30 minutes and then were processed for data collection on the BioRad ZE5 cytometer. Data was then analyzed using MODFITS. Experiments were replicated three times.

### 2.7 β-Galactosidase assays

For *in vitro* analysis, cells were seeded on a 6-well plate and given 24 hours to adhere. 100,000 cells per well were plated for single drug while cells treated with combination were plated at 200,000 cells per well to account for drug effects. Cells were then treated with ND, 0.1 µM of doxorubicin, 0.25 µM of doxorubicin, 25 µM of venetoclax, or 0.1 µM of doxorubicin in combination with 25 µM of venetoclax for 5-6 days. A lower dose of doxorubicin was selected given the longer time point of the experiment. Following drug exposure, cells were fixed and stained using a Senescence β-Galactosidase (β-Gal) Staining Kit (Cell Signaling Technology, Danvers, MA). Cells were then incubated at room temperature for 10 minutes in 300 nM of DAPI (ThermoFisher Scientific, Waltham, WA). Cells were imaged on an Olympus iX83 microscope to assess β-Gal expression. For *in vivo* analysis, tissue was collected from all sample groups on day 37 and frozen in optimal cutting temperature (OCT). OCT samples were cut on a cryostat at 5 µm then immediately fixed and stained with the previous kit described. Samples were then counterstained with eosin for 30 seconds, rinsed with water, and mounted with Permount (ThermoFisher Scientific, Waltham, MA). An Olympus iX83 microscope was utilized to obtain representative images at 20X magnification. β-Gal and Ki-67 quantification was performed using Image J analysis to calculate the percentage area of pixel density. Each experiment was replicated three times.

### 2.8 Immunoblotting

Cells were plated on 100 mm dishes and allowed to adhere for 24 hours. Cells were then dosed with ND, 0.5 µM of doxorubicin, 25 µM of venetoclax, or 0.5 µM of doxorubicin in combination with 25 µM of venetoclax. Following 24 hours of drug exposure, media was aspirated and collected. Media was centrifuged and cell pellets were collected with adherent cells on ice using RIPA lysis buffer (ThermoFisher Scientific, Waltham, MA). Cells were centrifuged at 2,500 rpm for 5 minutes and then sonicated for 30 seconds. Sonicated samples were then placed in a 4°C centrifuge and spun at >10,000 rpm for 10 minutes. Protein supernatant was then analyzed using Pierce BCA Protein Assay Kit (ThermoFisher Scientific, Waltham, MA). Protein supernatant was then combined with dye, reducing agent and complete lysis buffer (ThermoFisher Scientific, Waltham, MA). Samples were heated at 90°C for 7 minutes and then loaded into Invitrogen 4-12% Bis-Tris Midi 1.0 mm gradient gel and immersed in 1X MOPS Running Buffer (ThermoFisher Scientific, Waltham, MA). Gels underwent gel electrophoresis for ~1.5-2 hours and were then transferred to a nitrocellulose membrane using Thermo Scientific Pierce G2 Fast Blotter. Membranes were then exposed to blocking buffer on an orbital shaker for 1 hour. After an hour incubation, blocking buffer was removed and membranes were immersed in tween blocking buffer with primary antibodies overnight at 4°C. The next day, membranes were washed three times with 1X TBST and then exposed to secondary antibodies for one hour at room temperature. Membranes were then again washed three times with 1X TBST and analyzed using Odyssey Infrared Imaging System (LI-COR Biosciences, Lincoln, NE).

The following primary antibodies were used for analysis: p-RAD50 (#14,223), p-H2AX (#9718), cleaved caspase-3 (#9661), p53 (#48,818), Bcl-2 (#4223), mcl-1 (#4572), PARP (#9532), p21 (#2946), p16 (#80,772), BCL-XL (#2764), cyclin B1 (#4135), and actin (#58,169, #4970) (all from Cell Signaling Technology, Danvers, MA). Secondary antibodies included anti-rabbit IgG (H + L) (DyLight 680 Conjugate, #5470) and/or rabbit IgG (H+L) (DyLight 800 4X PEG Conjugate, #5151) at 1:10,000 dilution (Cell Signaling Technology, Danvers, MA). Multiple independent blots were used to evaluate protein expression and complete western blots can be viewed in **Supplementary Material** (**Supplementary Figure 1**). Western blot quantification was performed by densitometry using Image J. All experiments were done in triplicate.

### 2.9 Murine studies

Female athymic nude mice (Hsd: Athymic Nude-Foxn1nu) were purchased from Envigo (Indianapolis, IN). 100 µL MDA-MB-231 (5x10^6^) cells were mixed with a 1:1 ratio of DMEM media and cultrex (Cultrex PathClear BME, Type 3 from Bio-Techne) and at approximately 3 months old, mice were injected subcutaneously into bilateral flanks. Once tumors reached a volume of ~50-100 mm^3^ mice were dosed with either vehicle, doxorubicin 1.5 mg/kg intra-peritoneal (IP) once every week, venetoclax 50 mg/kg by oral gavage daily Monday-Friday, or a combination of doxorubicin and venetoclax. Tumor volumes ([tumor volume = length x width^2^] x 0.52) and weights were measured twice a week and recorded in Study Director (Studylog version 3.1.399.23, San Francisco, CA). At the end of study, formalin-fixed paraffin-embedded tumors were collected and stained for hematoxylin and eosin (H&E) and Ki-67 using the University of Colorado Cancer Center Pathology Shared Resource. Tumor volume graphs and specific growth rate (SGR) were calculated using GraphPad Prism 10 as described previously (41–43).

### 2.10 Animal care and ethics

Mice were checked daily for toxicity, given supplemental foods, and euthanized if health or tumor endpoint criteria were met. All murine studies were done in compliance with the University of Colorado Anschutz Medical Campus Institutional Animal Care and Use Committee in accordance with IACUC animal protocol #00021. Maximal tumor size permitted per IACUC was 2000mm^3^ or total tumors of 3000mm^3^. Maximal tumor size was not exceeded in this study.

### 2.11 Statistical analysis

Statistical evaluation was done using Graph Pad Prism 10. P=0.05 was the cut-off for statistical significance. One-way ANOVA with Tukey correction was used for comparisons. Error bars represent standard error of the mean (SEM). Figure legends indicate specific tests applied.

## 3 Results

### 3.1 Venetoclax has synergistic anti-proliferative activity in combination with doxorubicin across multiple TNBC cell lines

TNBC cell lines were dosed with doxorubicin to identify which cell lines were more resistant and which cell lines were less resistant to doxorubicin as previously demonstrated by Smoots et al. (22). Cell lines with an absolute IC_50_ of 0-100 nM were considered sensitive, cell lines with an absolute IC_50_ of 100-300 nM were considered to have intermediate resistance, and cell lines with an absolute IC_50_ > 300 nM were considered resistant. Cell lines that were resistant (CAL-120), intermediate (Hs 578T, MDA-MB-231), and sensitive (CAL-51) to doxorubicin were selected for further analysis. All selected cell lines were mesenchymal stem-like/mesenchymal MSL/M and harbored a mutation in p53 except for CAL-51 which was p53 WT.

**Figure 1:**
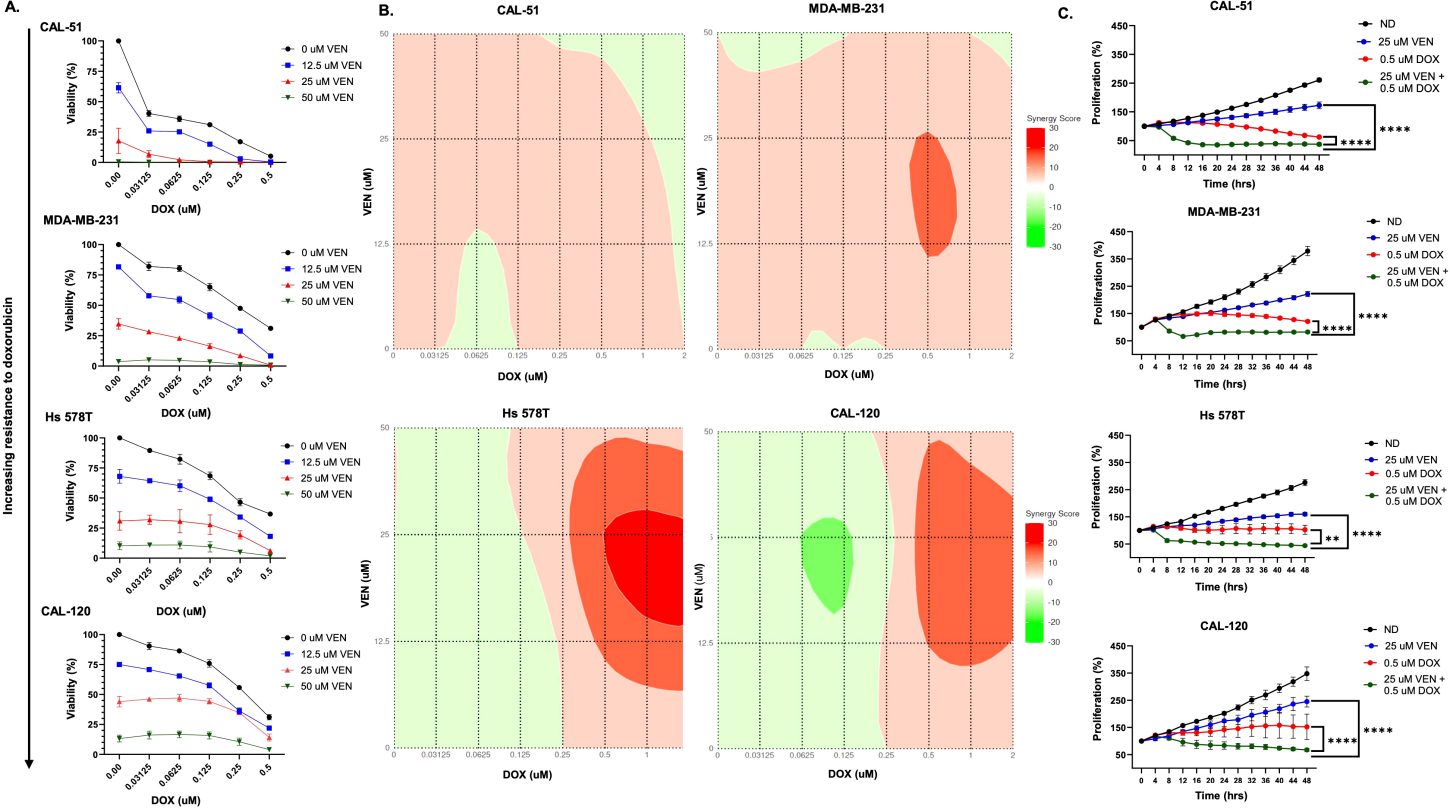
Doxorubicin + venetoclax has synergistic effects in TNBC cell lines. **(A)** Percentage of viable cells as measured by CellTiter-Glo after exposure to varying doses of doxorubicin (DOX) and venetoclax (VEN) for 72 hours. **(B)** Loewe synergy plots calculated from CellTiter-Glo analysis. **(C)** Anti-proliferative effects following 48 hours of dosing with no drug, 0.5 µM of DOX, 25 µM of VEN, or 0.5 µM of DOX in combination with 25 µM of VEN. Comparisons were performed using an ordinary one-way ANOVA with Tukey correction (**P < 0.01, ****P < 0.0001). Error bars represent standard error of the mean (SEM). All experiments were done in triplicate.

There was lower cellular viability in all cell lines treated with the combination of doxorubicin and venetoclax when compared to single agent doxorubicin or venetoclax (**Figure 1A**, **Supplementary Figure 2**). All cell lines reached an IC_80_ when treated with top doses of venetoclax and doxorubicin in combination (**Figure 1A**). Loewe and Bliss additivity models showed synergetic effects with combination dosing, which was most notable in the MDA-MB-231, Hs 578T, and CAL-120 cell lines (**Figure 1B**, **Supplementary Figure 3**). In the Loewe model, synergy scores ranged from -2 to 11 in the MDA-MB-231 cell line, -10 to 23 in the Hs 578T cell line, and -11 to 19 in the CAL-120 cell line. In the Bliss model, synergy scores ranged from -2 to 17 in the MDA-MB-231 cell line and -7 to 11 in the Hs 578T cell line. In all cell lines, the combination of venetoclax with doxorubicin resulted in a statistically significant decrease in proliferation when compared to single agent doxorubicin and venetoclax (**Figure 1C**, **Supplementary Figure 4**). The combination of doxorubicin with venetoclax also increased cell death in all cell lines (**Figure 1C**).

### 3.2 The combination of doxorubicin with venetoclax increases apoptosis

**Figure 2:**
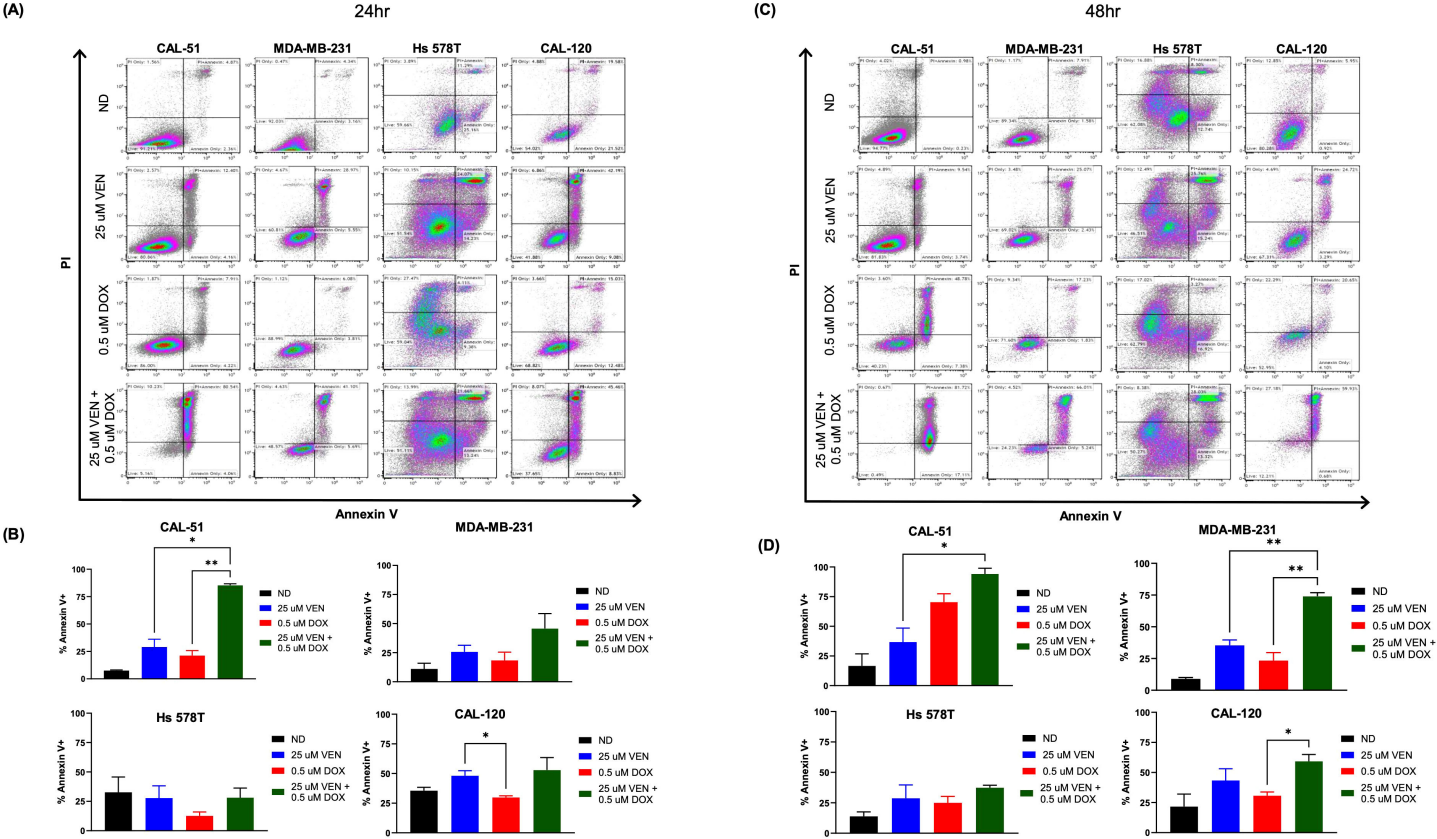
Apoptotic effects on TNBC cell lines. Cells were dosed with no drug (ND), 0.5 µM of doxorubicin (DOX), 25 µM of venetoclax (VEN), or 0.5 µM of DOX in combination with 25 µM of VEN. **(A)** Flow cytometry analysis of Annexin V/PI following 24 hours of drug exposure. **(B)** Percentage of total Annexin V+ following 24 hours of drug exposure. **(C)** Flow cytometry analysis of Annexin V/PI following 48 hours of drug exposure. **(D)** Percentage of total Annexin V+ following 48 hours of drug exposure. Comparisons were performed using an ordinary one-way ANOVA with Tukey correction. (*P < 0.05, **P < 0.01). Error bars represent standard error of the mean (SEM). All experiments were done in triplicate.

Using the Annexin-V/PI assay, we assessed for apoptosis and cell death following treatment with doxorubicin and venetoclax. Following 24 hours of combination drug exposure, increased apoptosis and cell death, as measured by PI and Annexin V staining, was observed in the CAL-51, MDA-MB-231, Hs 578T, and CAL-120 cell lines (**Figure 2A**). This increase was statistically significant in the CAL-51 cell line and trends to increased Annexin V+ in the MDA-MB-231 and CAL-120 cell lines (**Figure 2B**, **Supplementary Figure 5**). After 48 hours of drug exposure, CAL-51, MDA-MB-231, Hs 578T, and CAL-120 had a higher percentage of cells that stained positive for PI and Annexin V when treated with the combination of doxorubicin and venetoclax (**Figure 2C**). The MDA-MB-231 cell line demonstrated increased Annexin V+ following combination compared to single agents which was statistically significant in a subset of cell lines (**Figure 2D**, **Supplementary Figure 5**).

### 3.3 The addition of venetoclax to doxorubicin reduces cellular senescence and impacts the cell cycle

**Figure 3:**
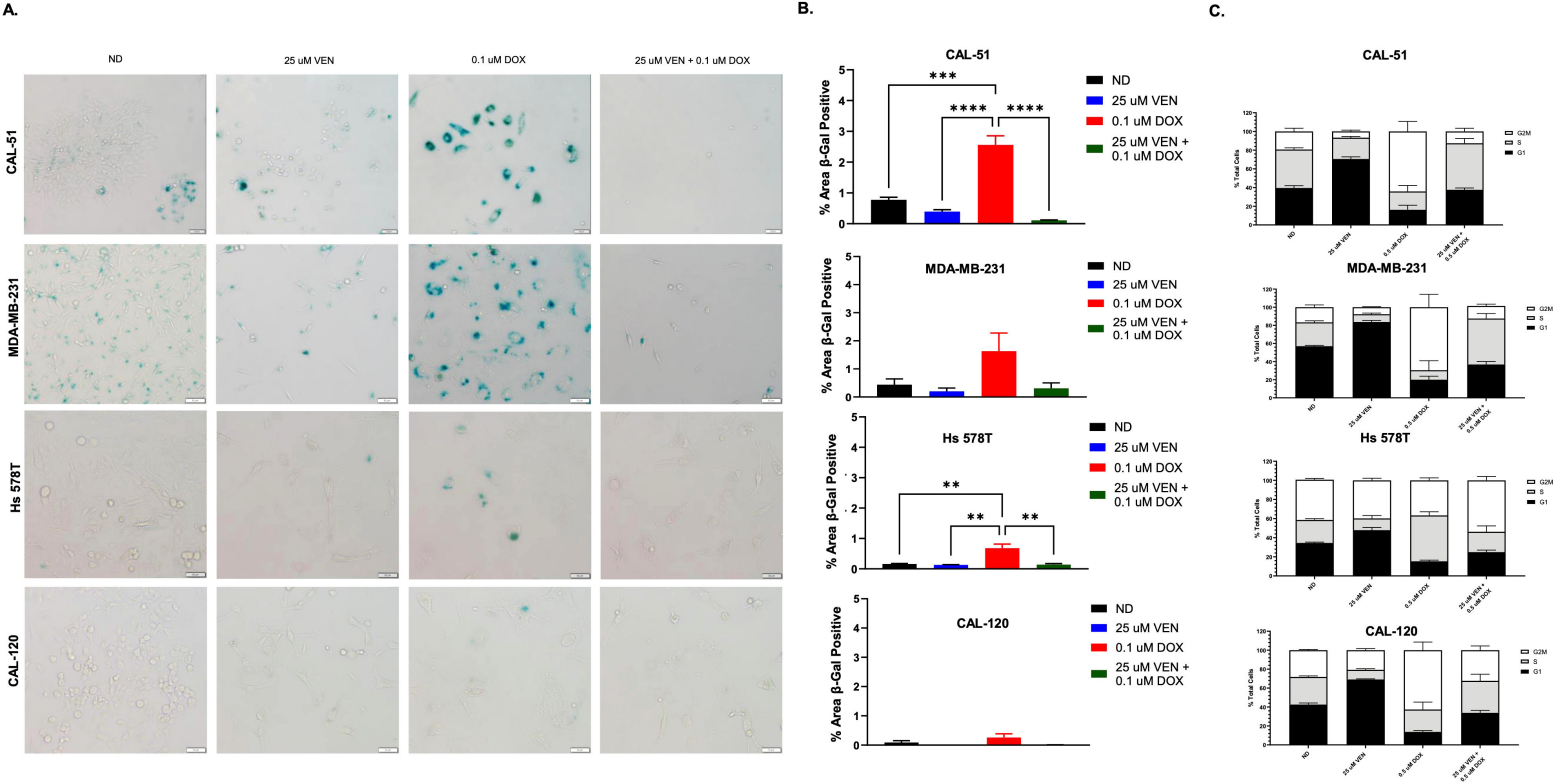
Senescence and cell cycle analysis of TNBC cell lines. **(A)** β-Gal staining indicating senescence following 6 days of treatment with no drug (ND), 0.1 µM of doxorubicin (DOX), 25 µM of venetoclax (VEN), or 0.1 µM of DOX in combination with 25 µM of VEN. **(B)** Percentage area of β-Gal positive cells. **(C)** Cell cycle analysis following 24 hours of exposure to ND, 0.5 µM of DOX, 25 µM of VEN, or 0.5 µM of DOX in combination with 25 µM of VEN. Comparisons were performed using an ordinary one-way ANOVA with Tukey correction. (**P < 0.01, ***P <0.001, ****P < 0.0001). Error bars represent standard error of the mean (SEM). All experiments were done in triplicate.

To assess for doxorubicin induced senescence, we performed the senescence-associated β-Gal assay after 6 days of doxorubicin exposure (**Figure 3A**). Across all assessed cell lines, β-Gal staining was the highest in cells treated with single agent doxorubicin at 0.1 µM when compared to the other treatment groups (ND, single agent venetoclax, and combination) (**Figure 3B**). Combination dosing with doxorubicin 0.1 µM and venetoclax 25 µM resulted in a reduction of β-Gal staining across all cell lines, though was most significant in the CAL-51 and Hs 578T cell lines (**Figure 3B**).

Following treatment with single agent doxorubicin, there was a significantly prolonged G2/M phase noted in the CAL-51, MDA-MB-231, and CAL-120 cell lines when compared to ND (**Figure 3C**, **Supplementary Table 1**). With the addition of venetoclax, the G2/M phase was shortened. Conversely, in the Hs 578T cell line, the G2/M phase was unchanged following treatment with single agent doxorubicin and was prolonged when treated in combination. There was a significantly prolonged G1 phase following treatment with single agent venetoclax across all cells line compared to ND (**Supplementary Table 1**). With combination dosing, the G1 phase was shortened.

### 3.4 Combination dosing impacts DNA damage and mediators of apoptosis

**Figure 4:**
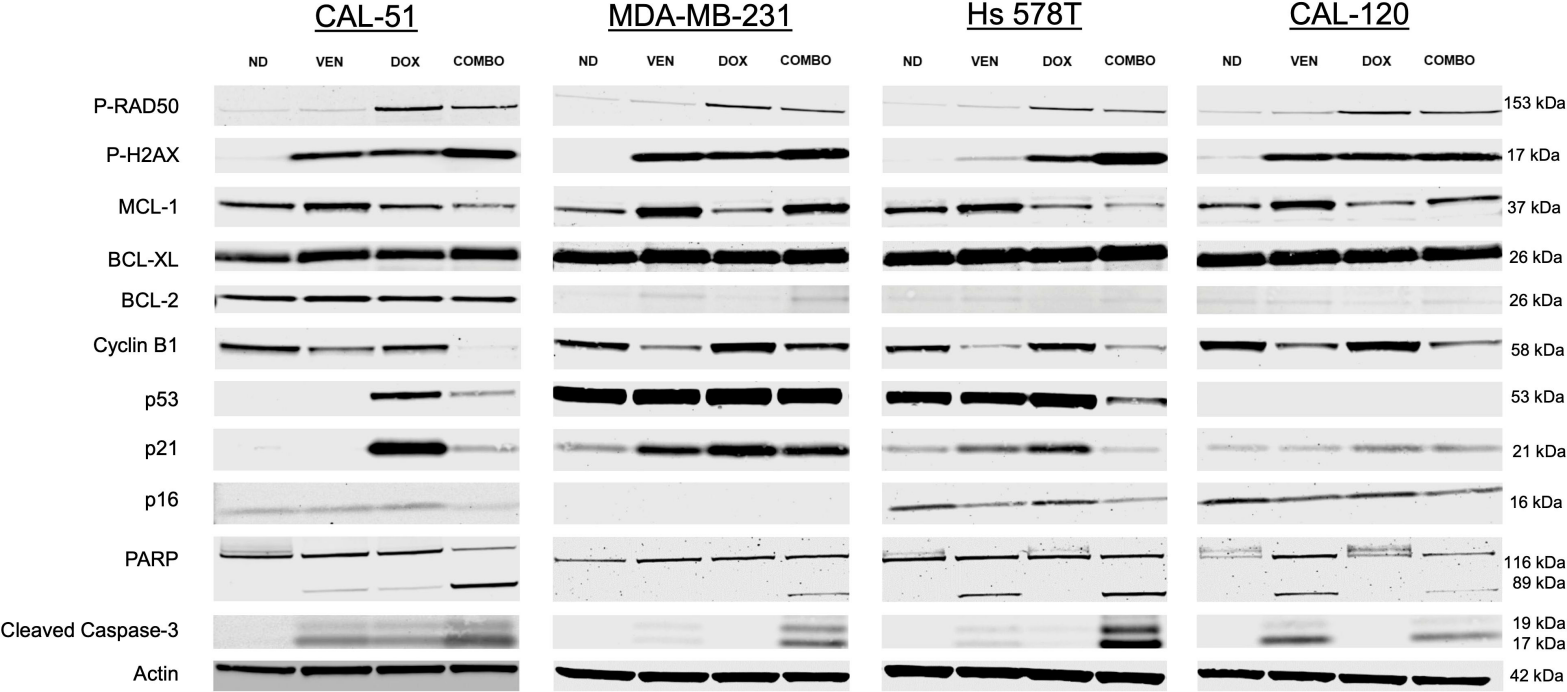
Western blot analysis. Downstream effects following 24 hours of treatment with no drug (ND), 0.5 µM of doxorubicin (DOX), 25 µM of venetoclax (VEN), or 0.5 µM of DOX in combination with 25 µM of VEN (COMBO) in CAL-51, MDA-MB-231, Hs 578T, and CAL-120 TNBC cell lines. All experiments were done in triplicate. kDa: kilodalton.

We assessed for markers of apoptosis, cell cycle checkpoints, and senescence following 24 hours of treatment with doxorubicin and venetoclax. Across all cell lines, there was evidence of DNA damage (increase P-RAD50 expression) in cells dosed with single agent doxorubicin and in combination (**Figure 4**). In all cell lines except CAL-120, there was evidence of cell cycle arrest and repair (increase P-H2AX) in combination dosing. The anti-apoptotic protein MCL-1 was down-regulated in CAL-51 and Hs 578T cells treated in combination. These effects on MCL-1 were not observed in the MDA-MB-231 and CAL-120 cell lines. BCL-2 appeared unchanged in CAL-51 and CAL-120 cell lines, however, was upregulated in MDA-MB-231 and Hs 578T cells treated with single agent venetoclax and in combination. There was a higher ratio of BCL-2 to BAX across all cell lines when dosed in combination (**Supplementary Figures 6A, B**). CAL-51 had a more pronounced reduction in cyclin B1 expression when treated in combination when compared to either single agent. This reduction was also observed in the other cell lines treated in combination, though cyclin B1 had similar expression following treatment with single agent venetoclax. Higher p53 expression was observed following treatment with single agent doxorubicin in CAL-51 and there was a lower expression of p53 in Hs 578T cells treated in combination when compared to either single agent. p53 expression was unchanged across treatment groups in MDA-MB-231 cells and was absent in CAL-120 due to the specific mutation in p53. p21 expression was more prominent in cells dosed with single agent doxorubicin in the CAL-51 and Hs 578T cell lines. In the MDA-MB-231 and CAL-120 cell lines, p21 expression was similar in single agent and combination groups. p16 expression was down-regulated in the combination group in the CAL-51 and Hs 578T cell lines. There was a higher expression of PARP and cleaved caspase-3 in CAL-51, MDA-MB-231, and Hs 578T cells treated in combination. In CAL-120, there was a higher expression of PARP and cleaved caspase-3 when compared to no drug and doxorubicin, but not when compared to venetoclax. The ratio of cleaved-PARP to PARP was calculated showing more cleaved-PARP expression in the CAL-51, MDA-MB-231, and Hs 578T cell line (**Supplementary Figure 7**).

### 3.5 Loss of p53 does not diminish the combination effects of doxorubicin and venetoclax

**Figure 5:**
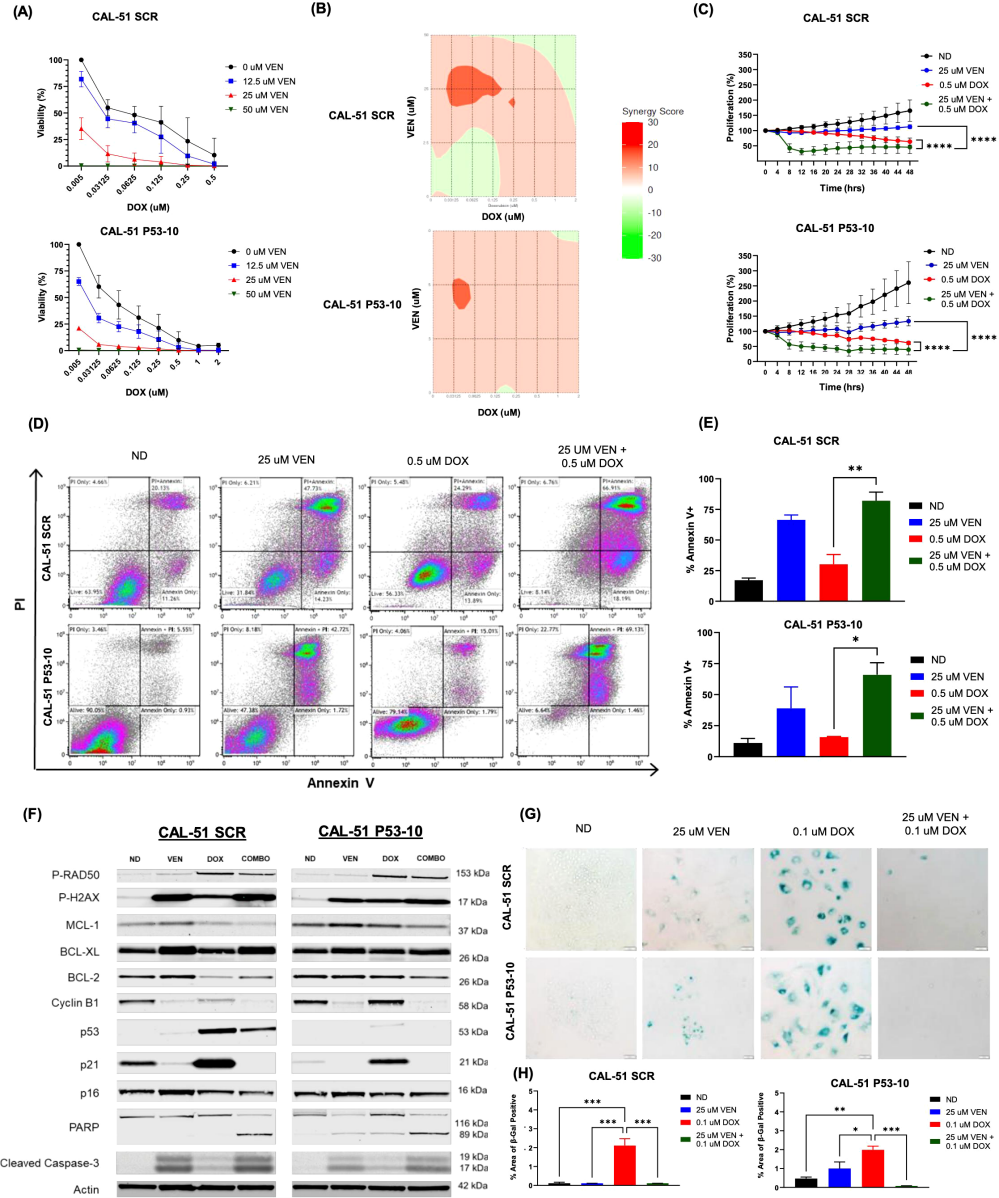
Synergistic and apoptotic effects in CAL-51 p53 knock-down and scramble cell lines. **(A) **Percentage of viable cells using CellTiter-Glo analysis after 72 hours of drug exposure to varying doses of doxorubicin (DOX) and venetoclax (VEN). **(B)** Loewe synergy plots calculated from CellTiter-Glo analysis. **(C)** Anti-proliferative effects following 48 hours of dosing with no drug (ND), 0.5 µM of DOX, 25 µM of VEN, or 0.5 µM of DOX in combination with 25 µM of VEN. **(D)** Flow cytometry analysis of Annexin V/PI following 24 hours of drug exposure. Cells were dosed with ND, 0.5 µM of DOX, 25 µM of VEN, or 0.5 µM of DOX in combination with 25 µM of VEN. **(E)** Percentage of total Annexin V+ following 24 hours of drug exposure. Cells were dosed with ND, 0.5 µM of DOX, 25 µM of VEN, or 0.5 µM of DOX in combination with 25 µM of VEN. **(F)** Western blot analysis of downstream effects following 24 hours of treatment with ND, 0.5 µM of DOX, 25 µM of VEN, or 0.5 µM of DOX in combination with 25 µM of VEN (COMBO). **(G)** β-Gal staining indicating senescence following 6 days of treatment with ND, 0.1 µM of DOX, 25 µM of VEN, or 0.1 µM of DOX in combination with 25 µM of VEN. **(H)** Percentage area of β-Gal positive cells. Comparisons were performed using an ordinary one-way ANOVA with Tukey correction (*P < 0.05, **P < 0.01, ***P <0.001, ****P < 0.0001). Error bars represent standard error of the mean (SEM). All experiments were done in triplicate.

CAL-51 p53 SCR and CAL-51 p53-10 KD down cell lines treated with doxorubicin and venetoclax demonstrated synergy and a reduction in cellular viability and proliferation (**Figures 5A–C**, **Supplementary Figures 8A, B**). Loewe synergy scores in CAL-51 p53 SCR and CAL-51 p53-10 KD ranged from -6 to 14 and -0.4 to 11, respectively, also indicating a synergistic interaction (**Figure 5B**). Bliss synergy scores in CAL-51 p53 SCR and CAL-51 p53-10 KD ranged from -1 to 11 and -0.1 to 9, respectively, indicating synergistic anti-proliferative effects (**Supplementary Figure 9** **).** Annexin V expression increased following combination treatment and was significant in both CAL-51 p53-10 KD and CAL-51 p53 SCR when compared to single agent doxorubicin (**Figures 5D, E**, **Supplementary Figure 10**). DNA damage was noted in both cell lines treated in combination and with single agent doxorubicin as noted by the higher expression of P-RAD50 (**Figure 5F**). Expression of P-H2AX was also upregulated in combination dosing in both cell lines (**Figure 5F**, **Supplementary Figure 11A**). There was evidence of cell cycle disruption in both cell lines treated in combination indicated by lower expression of cyclin B1 (**Figure 5F**, **Supplementary Figure 11B**). There was lower expression of the anti-apoptotic protein MCL1 in combination treatment in both CAL-51 p53 SCR and CAL-51 p53 KD cell lines. BCL-2 expression was lowest in the doxorubicin treated cells in the CAL-51 p53 SCR cell line. There was a higher ratio of BCL-2 to BAX across both cell lines when dosed in combination (**Supplementary Figure 12**). BCL-XL had higher expression following treatment with single agent venetoclax and in combination in the CAL-51 p53 SCR cell line. In both cell lines, p21 demonstrated increased expression following treatment with single agent doxorubicin and had lower expression following treatment with combination when compared to single agent doxorubicin (**Figure 5F**, **Supplementary Figure 11C**). There were no significant changes in p16 expression in both cell lines across treatment groups. There was evidence of more apoptosis in both cell lines treated in combination with an increase in PARP and cleaved caspase-3 (**Figure 5F**). In both cell lines, treatment with single agent doxorubicin resulted in higher levels of β-Gal staining which was reduced with the combination treatment of doxorubicin with venetoclax (**Figures 5G, H**).

### 3.6 The combination of doxorubicin with venetoclax reduces β-Galactosidase staining *in vivo*

**Figure 6:**
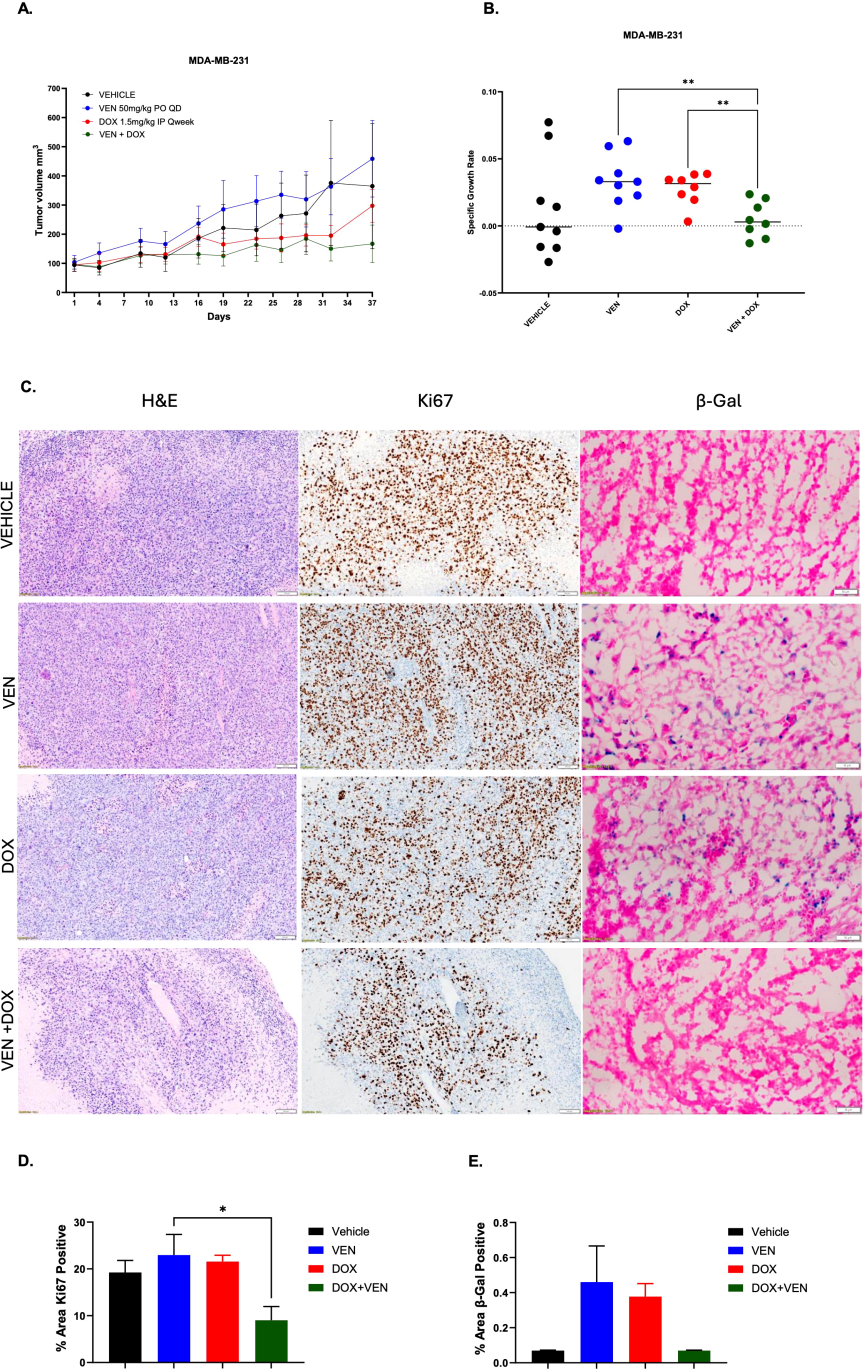
Assessment of the combination of doxorubicin + venetoclax *in vivo.* **(A)** Tumor volumes of MDA-MB-231 murine models treated with vehicle, doxorubicin (DOX), venetoclax (VEN) or combination over 37 days. **(B)** SGR at day 37 depicting overall tumor growth inhibition. **(C)** Staining for H&E, Ki-67, and β-Gal on frozen tumor tissue collected at end of treatment, day 37. Representative images were taken at 20x magnification. Scale for H&E and Ki-67 is 100 µm. Scale for β-Gal is 50 µm. **(D)** Percentage area of Ki-67 positive cells. **(E)** Percentage area of β-Gal positive cells. Comparisons were performed using an ordinary one-way ANOVA with Tukey correction (*= P < 0.05, **P < 0.01). There were 8-9 sub-cutaneous tumors per group with tumor volumes ~50-100mm^3^.

The impact of doxorubicin in combination with venetoclax was assessed *in vivo*. The combination of doxorubicin with venetoclax resulted in enhanced tumor growth inhibition when compared to single agents in murine models injected with MDA-MB-231 cells (**Figure 6A**, **Supplementary Figure 13**). There was a significant reduction in SGR in the combination group when compared to both single agents (**Figure 6B**). Tumor analysis done at the end of treatment indicated that tumor cells treated in combination had less cellularity and lower rates of proliferation (**Figures 6C, D**). In addition, there was less β-Gal staining in tumors treated in combination when compared to single agent drugs (**Figures 6C, E**). Combination dosing resulted in a lower net body weight percentage when compared to single agents, however this was managed with supplemental foods daily and none of the mice had to be euthanized (**Supplementary Figure 14**).

## 4 Discussion

Anthracyclines are an integral part of the treatment of TNBC. Overcoming anthracycline resistance is an important step toward improving outcomes in patients diagnosed with TNBC. The purpose of this study was to assess the combination of venetoclax with doxorubicin and evaluate the potential to overcome anthracycline-induced senescence. In addition, we sought to determine the role of p53 in relation to senescence.

In both p53 WT, mutant, and KD TNBC cell lines, the combination of venetoclax with doxorubicin had synergistic effects and resulted in a significant decrease in cell growth and an increase in necrosis and apoptosis when compared to single agent venetoclax and single agent doxorubicin. This combination appeared effective regardless of p53 and had an impact on all assessed cell lines, even in the cell line most resistant to doxorubicin, CAL-120. The literature has demonstrated that the combination of doxorubicin and BH3 mimetics has synergistic effects and can cause an increase in cell death both *in vitro* and *in vivo* in a subset of TNBC cell lines (17, 37–39). Similar to these studies, we also found synergistic effects *in vitro* in the MDA-MB-231 and CAL-51 cell lines. Compared to other studies however, we demonstrate that there is a reduction of senescence *in vivo* following combination treatment and we show that the combination of venetoclax with doxorubicin had synergistic effects in an expanded panel of TNBC cell lines with varying resistance to doxorubicin and differing p53 mutational status, including p53 KD. This is important as *TP53* is commonly mutated in TNBC and it is crucial to have treatments that work regardless of p53 function. From previous research, it is known that p53 can drive tumor growth via loss-of-function, dominant-negative-effects, and gain-of-function (GOF) mutations (44–47). Wang et al. demonstrated that removing p53 with GOF mutations did not impact cellular response to chemotherapy and survival. This supports our findings, as we also did not find that p53 impacted cellular senescence or response to the combination dosing of venetoclax with doxorubicin.

As evidenced by β-Gal staining, we observed that doxorubicin induced a state of cellular senescence which was reduced when treated with the combination of doxorubicin with venetoclax. This was noted in all analyzed cell lines, though was less prominent in CAL-120. We also evaluated for other “hallmark” findings of senescence including a prolonged G1 or G2 phase of the cell cycle and over-expression of p21 and p16 (48–50). In mutant and WT p53 cell lines, there was an increase in cells in the G2/M phase following treatment with single agent doxorubicin. When treated with the combination of doxorubicin and venetoclax, the G2/M phase decreased dramatically, suggesting that venetoclax with doxorubicin eliminates senescent cells. Interestingly, this result was not appreciated in the Hs 578T cell line. In addition, over-expression of p21 and p16 was most prominent in CAL-51 and Hs 578T cells treated with single agent doxorubicin and expression diminished in combination. The MDA-MB-231 cell line has a mutation in CKDN2A and thus does not express p16. Notably, there was not a reduction of p21 expression in the MDA-MB-231 and CAL-120 cells that were treated in combination. This suggests that the mechanism of cellular apoptosis proceeds down a different pathway in these cell lines. In the CAL-51 SCR and KD cell lines, expression of p16 seemed unchanged, but p21 expression was over-expressed following treatment with single agent doxorubicin and under-expressed when treated in combination. We also found that when all cell lines were dose in combination, there was a higher ratio BCL-2 to BAX ratio. Studies have demonstrated that a lower BCL-2 to BAX ratio may suggest that cells are more susceptible to apoptosis (51, 52). It is possible that our results differ due to the 24-hour timepoint, and that at an earlier or later timepoint, the ratio would differ. It is also possible that the apoptosis we witnessed is not driven by BCL-2 and BAX. Taken together, our findings suggest that cellular senescence and elimination of senescence is not p53 mediated. Other studies have also suggested that senescence can occur independent of p53 (50, 53–56).

As noted above, there were some differences in cell lines and standard markers for senescence. CAL-120 did not robustly stain for β-Gal but had an increase in G2/M when treated with single agent doxorubicin as well as increase in PARP and cleaved caspase-3 when treated in combination. It is possible that in the CAL-120 cell line the mechanism of resistance was not senescence driven. Despite this, there was still evidence that the combination of doxorubicin with venetoclax resulted in more apoptosis in this cell line. The Hs 578T cell line did not have a shortened G2/M phase following treatment with combination but did have more staining with β-Gal when treated with single agent doxorubicin and had a notable reduction of p21 with combination treatment. Bojko et al. analyzed difference markers of senescence and did not find clear correlations – suggesting that each marker of senescence has limitations and that in order to best evaluate for senescence, multiple markers should be explored (49, 53). In addition, the Hs 578T cell line did not have a notable increase in Annexin V+ when treated in combination but did have other markers of apoptosis such as an increase in PARP and cleaved caspase-3 when treated in combination. It is possible that apoptosis and changes in the G2/M phase occur at different time-points and therefore were not captured by a 24 hour Annexin V/PI assay and cell cycle analysis.

We also evaluated for senescence in an *in vivo* mouse model. *In vivo* analysis demonstrated that there were enhanced anti-proliferative effects when doxorubicin was used in combination with venetoclax, a finding also supported in the work done by Inao et al. (38). Our data found that this combination of drugs resulted in the reduction of cellular senescence *in vivo*. Targeting doxorubicin induced senescence has potential and has been evaluated in combination with other senolytic agents *in vivo* (22). This combination has promise and should continue to be investigated with further *in vivo* work.

Clinically, there have been trials assessing the efficacy of BCL-2 inhibitors in combination with other anti-cancer agents in breast cancer. Currently, there is an on-going Phase I clinical trial investigating Olaparib (a PARP inhibitor) with navitoclax (BCL-2/BCL-XL inhibitor) in women with TNBC or high grade serous epithelial ovarian cancer who harbor germline mutations in *BRCA1/2* or *PALB2* (NCT05358639). Outside of TNBC, BCL-2 inhibitors have been explored in HR-positive breast cancers with mixed results. In a Phase Ib study evaluating the combination of venetoclax with tamoxifen in estrogen receptor (ER)-positive, BCL-2 positive, metastatic breast cancer, promising anti-tumor activity was noted (57). Recently the VERONICA trial evaluated the combination of fulvestrant with venetoclax in ER-positive metastatic breast cancer. The study did not meet its primary endpoint, though there was a non-significant increase in the clinical benefit rate (CBR) and progression free survival (PFS) in tumors that had over-expression of BCL-2 and BCL-2/BCL-XL H-score ratio of ≥1 (58). A preclinical study evaluating BCL-2 inhibitors and chemotherapy in breast cancer xenograft models demonstrated that BCL-2 targeted agents are more efficacious when used in combination with chemotherapy, which possibly explains the outcome of the VERONICA trial (59). Overall, the combination of doxorubicin with venetoclax is encouraging, however further *in vivo* investigation is warranted prior to clinical application.

There were several limitations to this study. While many “classic” markers of senescence were explored, further work could be done to further validate these findings. Future work could include a more comprehensive investigation of senescence-related genes such as IL6, IL8, and MMP3. In addition, to further define the mechanistic role and its relation to p53, p21, p16 and RB functional knockouts could be investigated. Furthermore, our mouse study used a nude mouse model, which did not allow examination of immune cell characteristics. Future work with an immunocompetent mouse model would be useful to investigate how this treatment combination impacts the immune system. Another limitation to our study is that the *in vitro* dose of venetoclax correlates to a higher plasma concentration than what is typically applied in clinic. Lower *in vitro* doses of venetoclax could be further explored in future studies. The *in vivo* dose of venetoclax however, did correlate to clinically relevant doses. Despite this, a mouse study devoted to examining the toxicity and pharmacokinetics of the combination would still be imperative prior to translation to a clinical setting.

## 5 Conclusion

In this study, we demonstrate that venetoclax can impact doxorubicin-induced cellular senescence and result in apoptosis. The combination of venetoclax with doxorubicin was effective in numerous TNBC cell lines, including those more resistant to doxorubicin, and those who had mutant, WT, or KD p53. We found that intact p53 is not required to push cells from a state of senescence to a state of apoptosis. In addition, the combination of doxorubicin with venetoclax was effective at eliminating senescent cells *in vivo.* The combination of venetoclax with doxorubicin represents an exciting avenue to overcome doxorubicin resistance and should continue to be explored.

## Data Availability

The original contributions presented in the study are included in the article/**Supplementary Material**. Further inquiries can be directed to the corresponding author.
