# Supplementary material for: Potentiating doxorubicin activity through BCL-2 inhibition in p53 wild-type and mutated triple-negative breast cancer
Source: Front Oncol. 2025 Apr 2;15:1549282. doi: 10.3389/fonc.2025.1549282 (PMC11999952; doi:10.3389/fonc.2025.1549282)

Supplemental Figure 1: Complete Western Blots

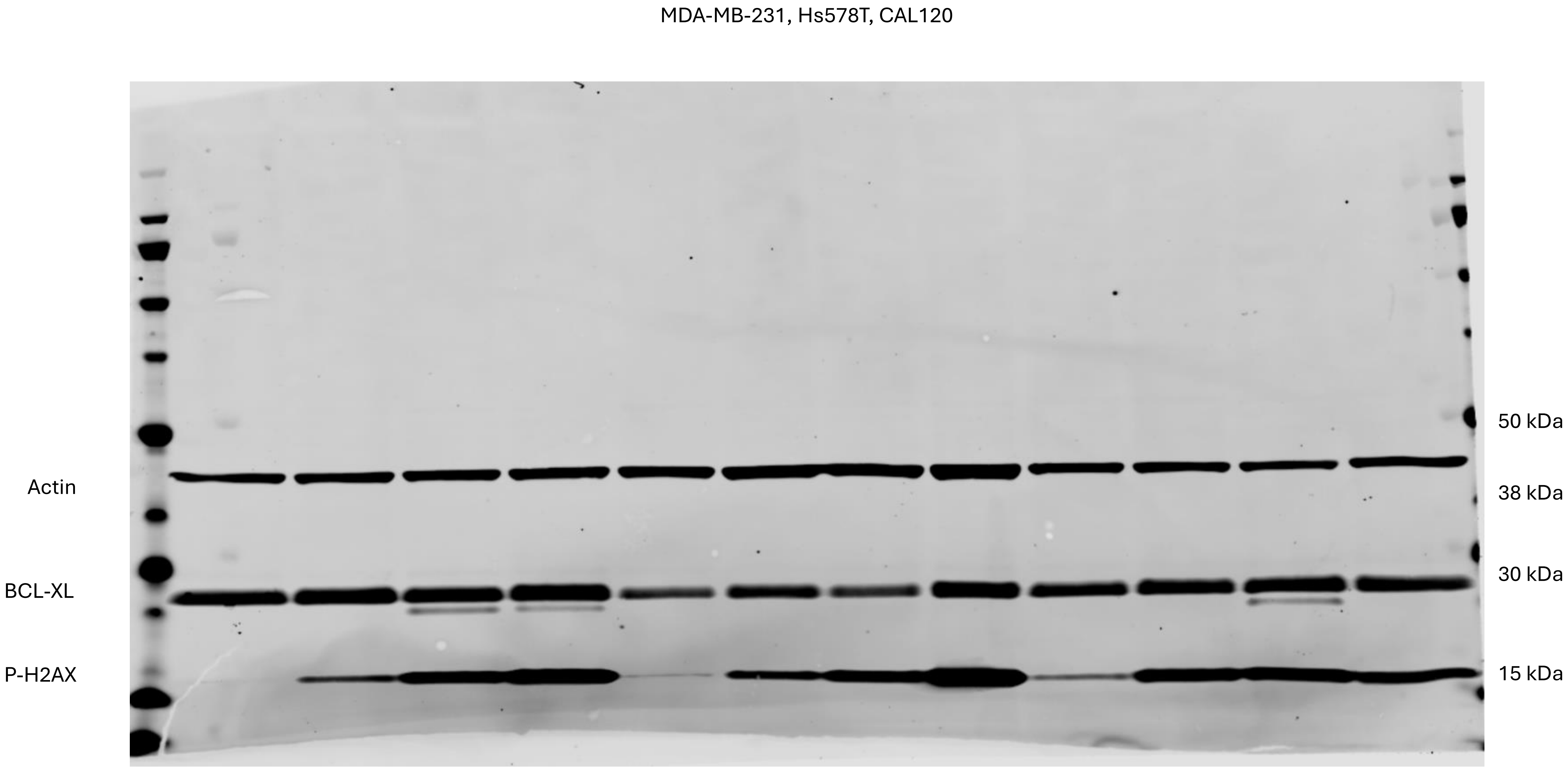

MDA-MB-231, Hs578T, CAL120

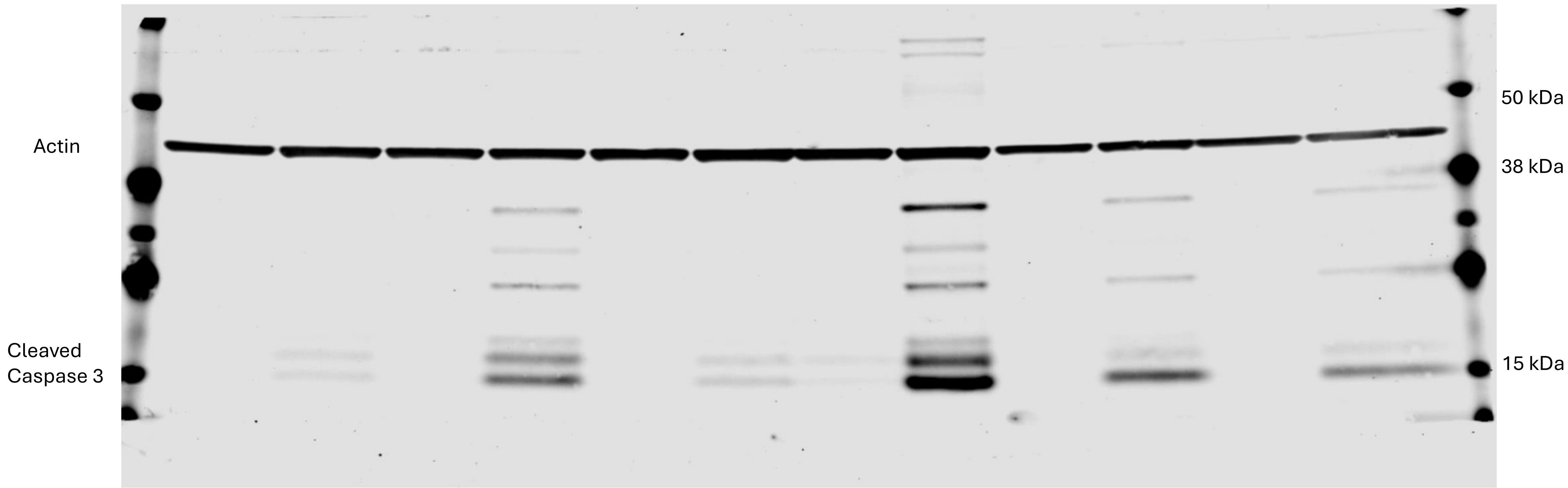

MDA-MB-231, Hs578T, CAL120

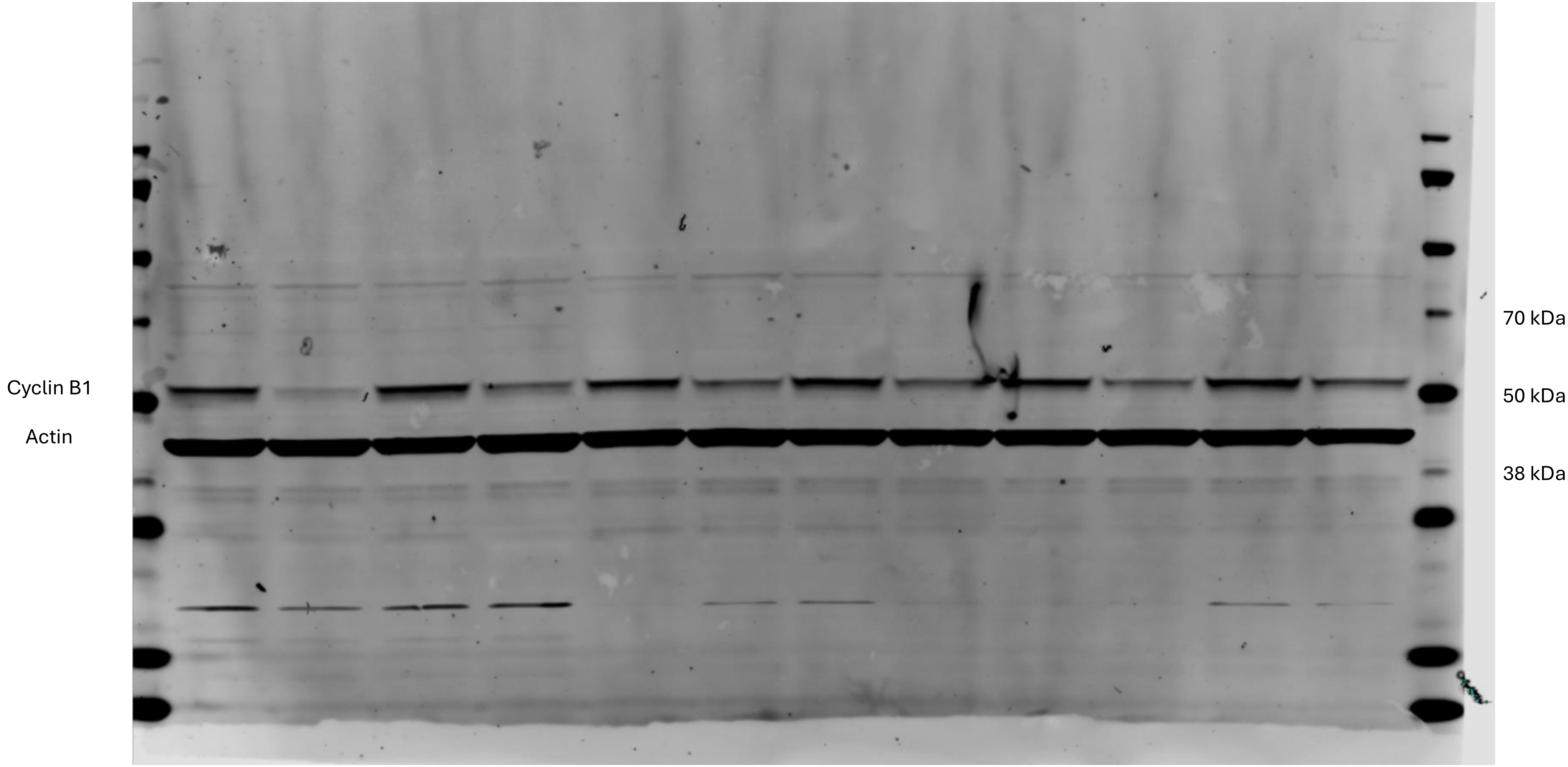

MDA-MB-231, Hs578T, CAL120

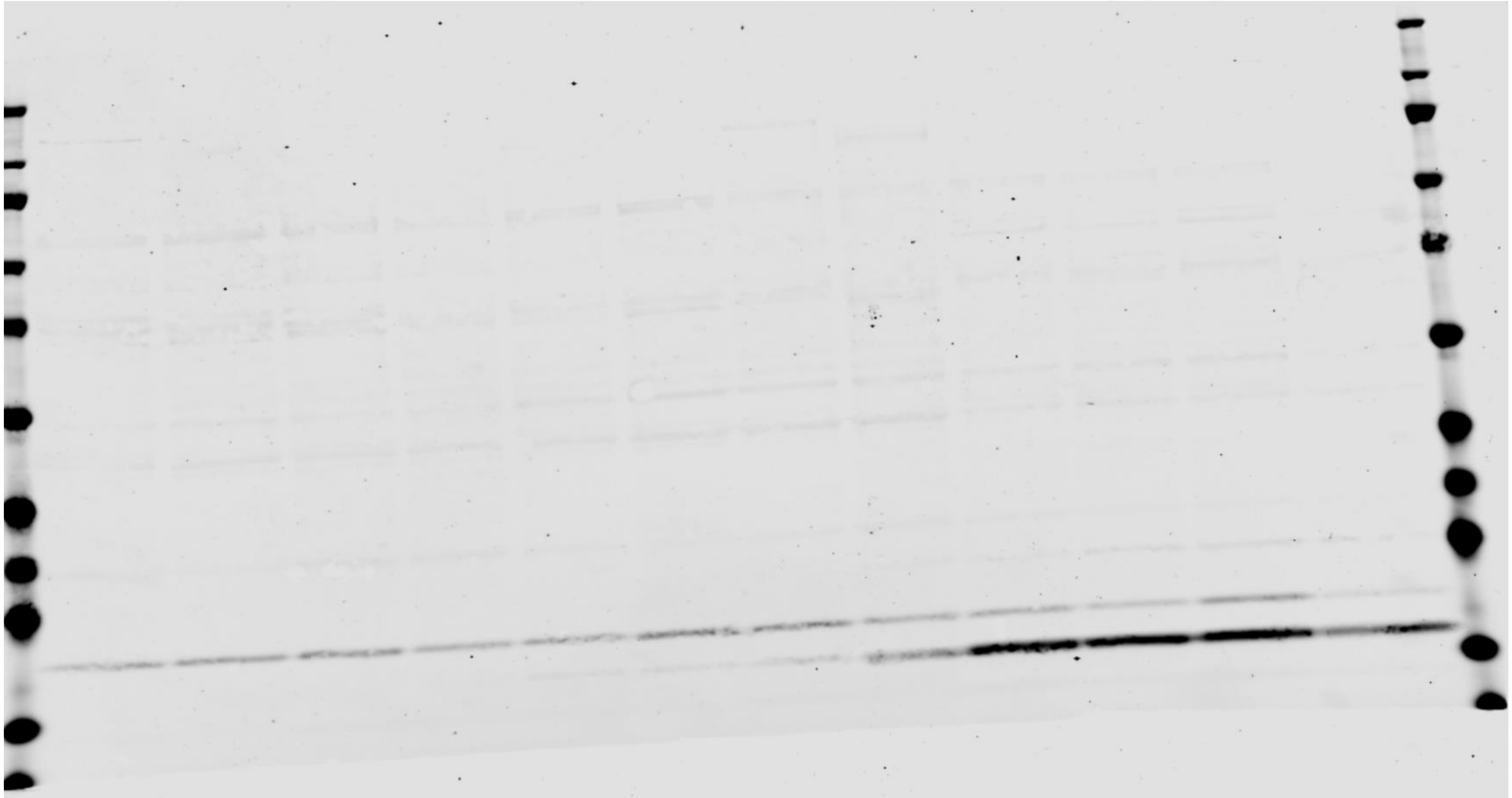

25 kDa

15 kDa

p16

MDA-MB-231, Hs578T, CAL120

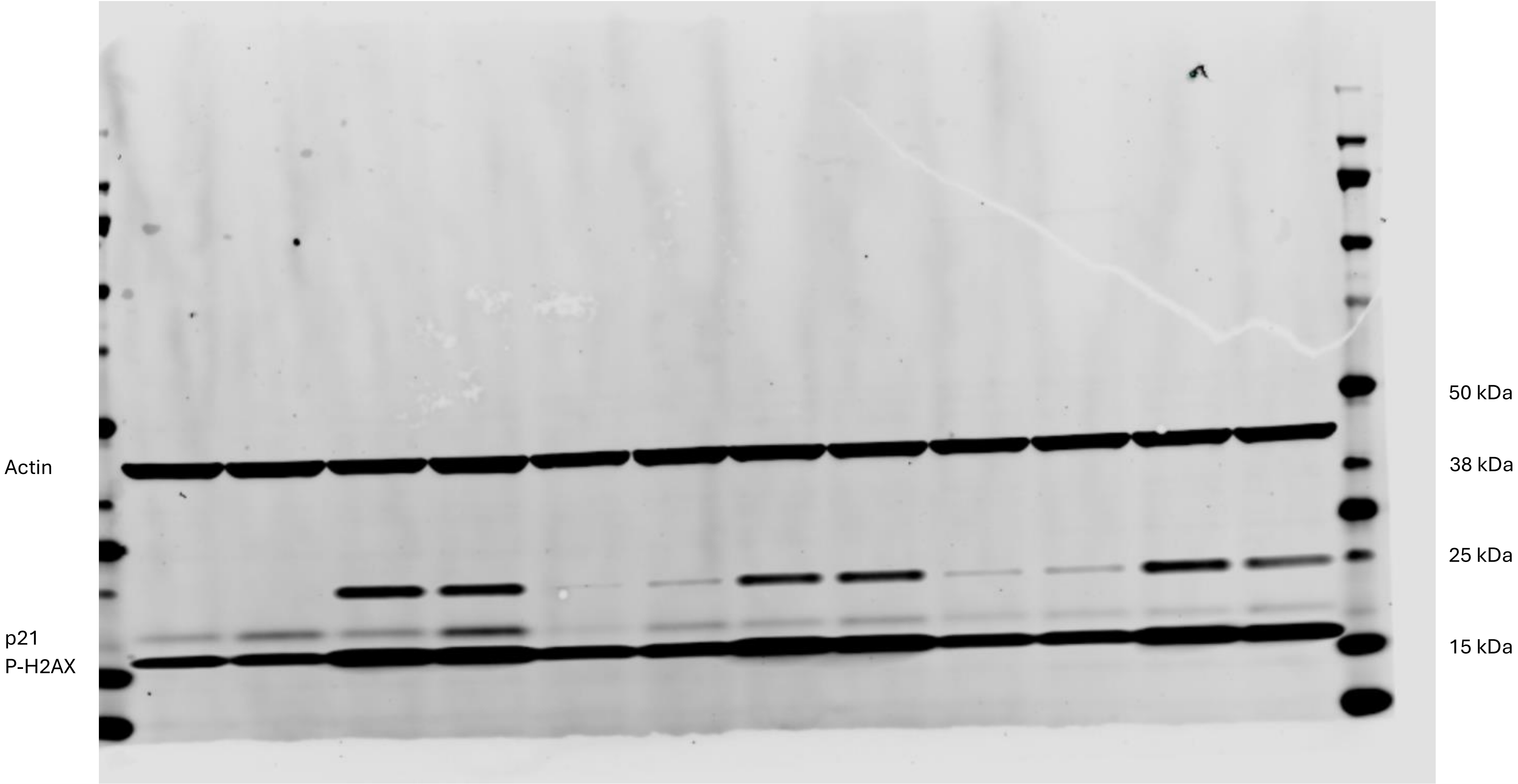

MDA-MB-231, Hs578T, CAL120

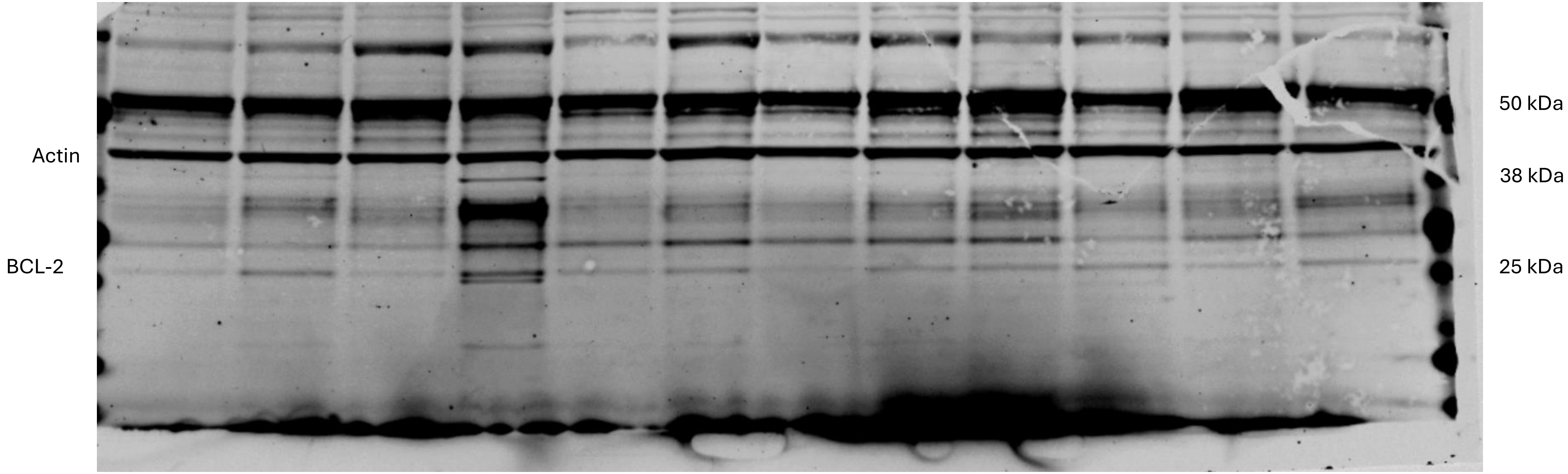

MDA-MB-231, Hs578T, CAL120

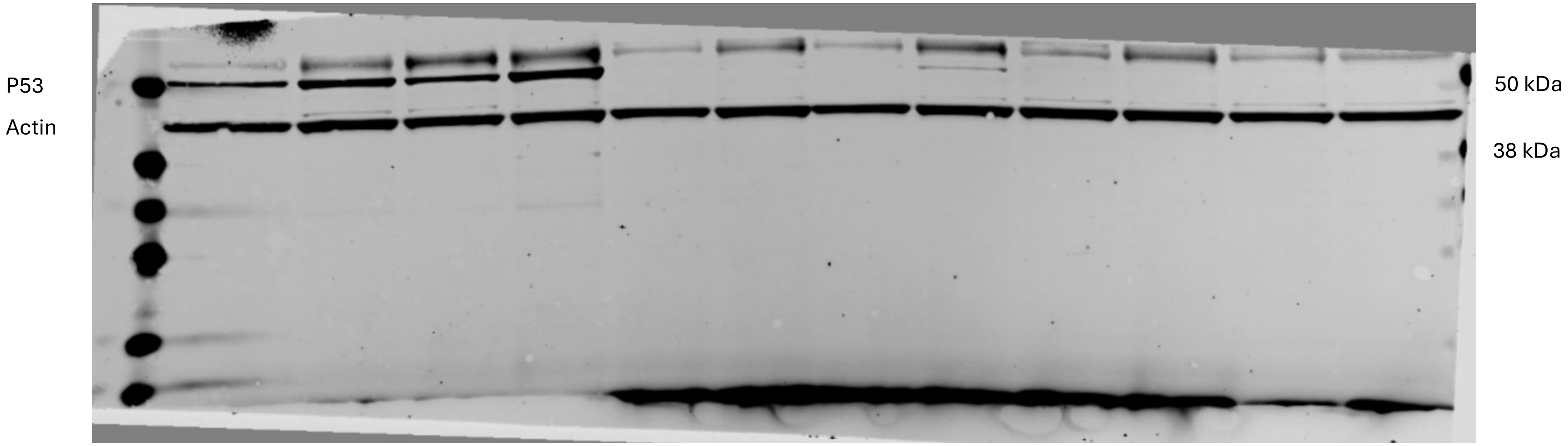

MDA-MB-231, Hs578T, CAL120

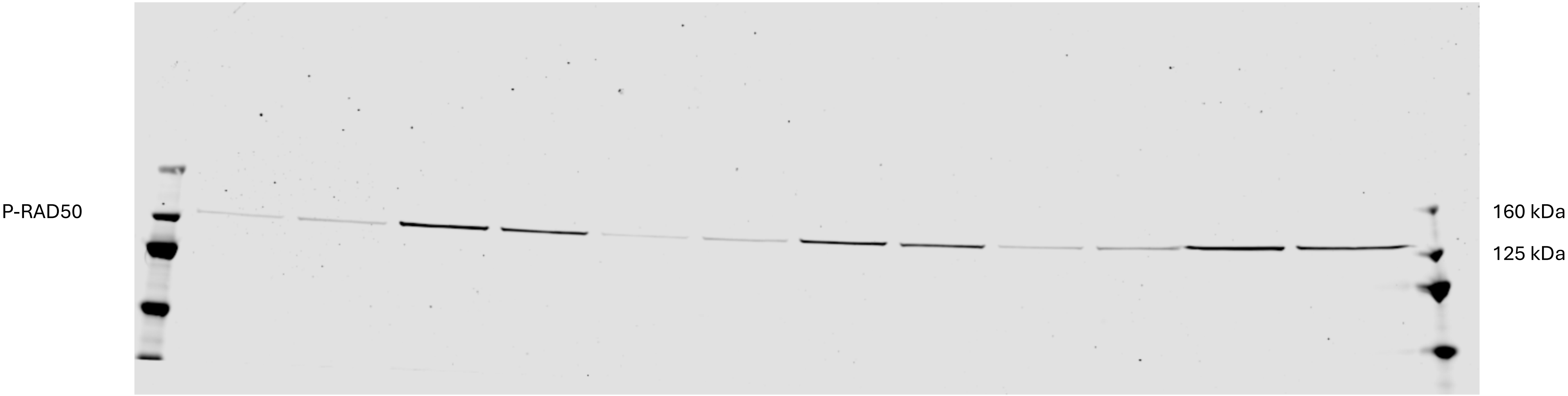

MDA-MB-231, Hs578T, CAL120

PARP

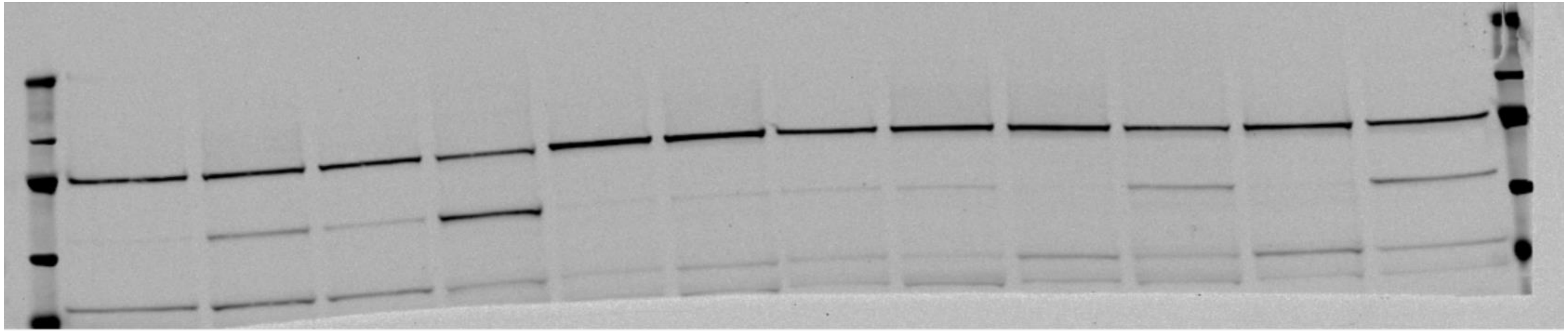

125 kDa

90 kDa

MDA-MB-231, Hs578T, CAL120

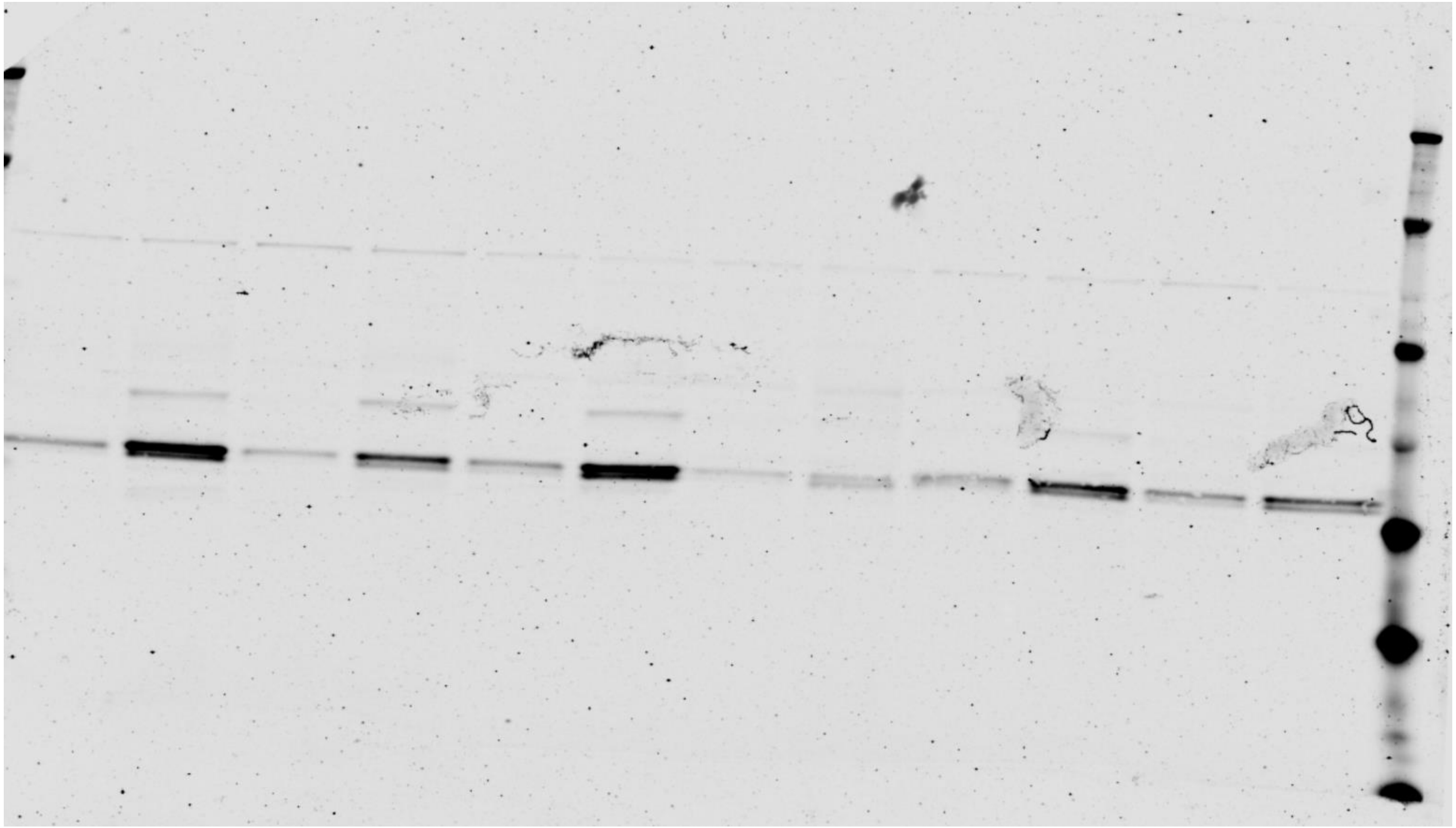

CAL51, SCR, P53-10

Actin

BCL-2

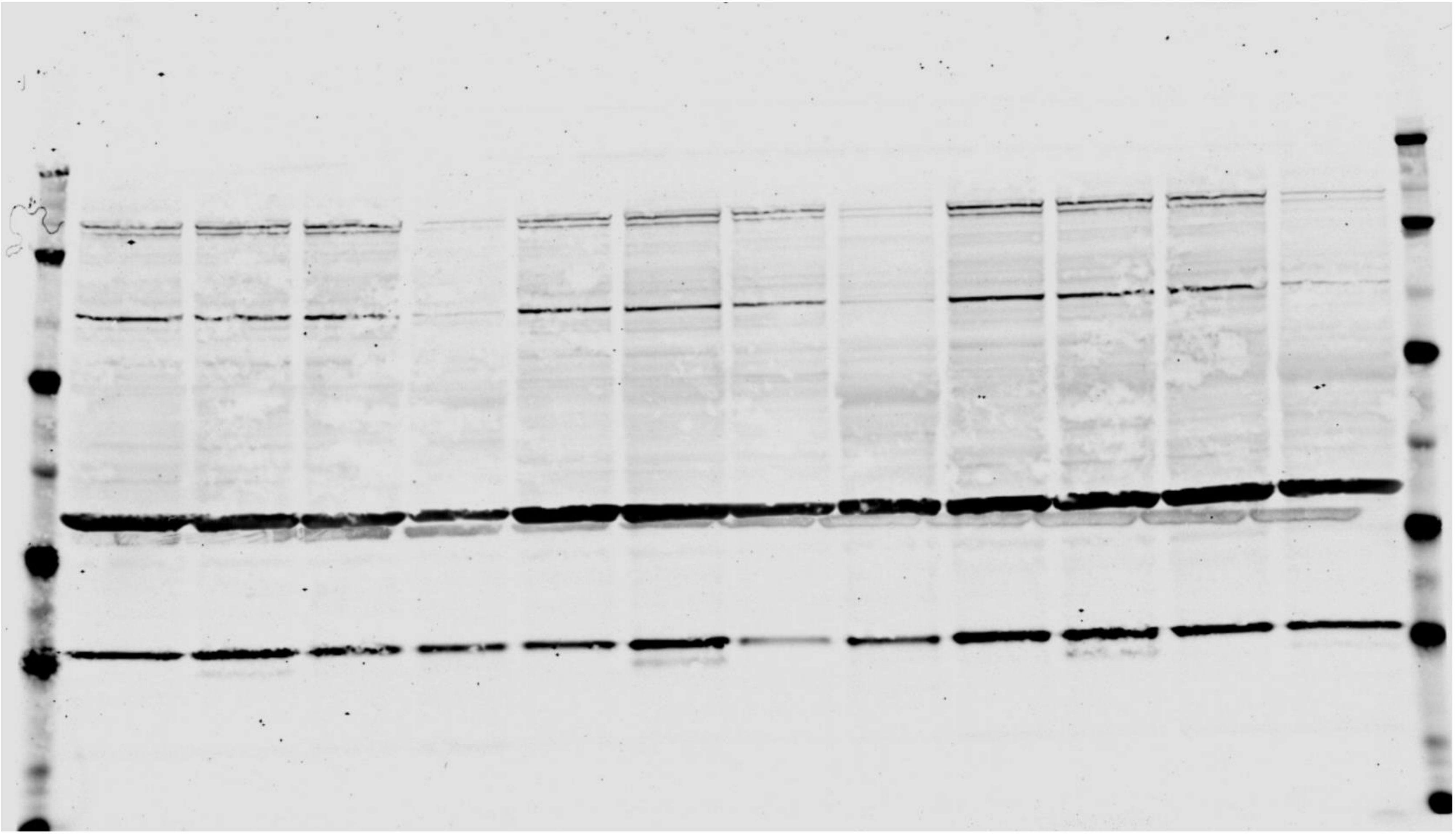

38 kDa

25 kDa

CAL51, SCR, P53-10

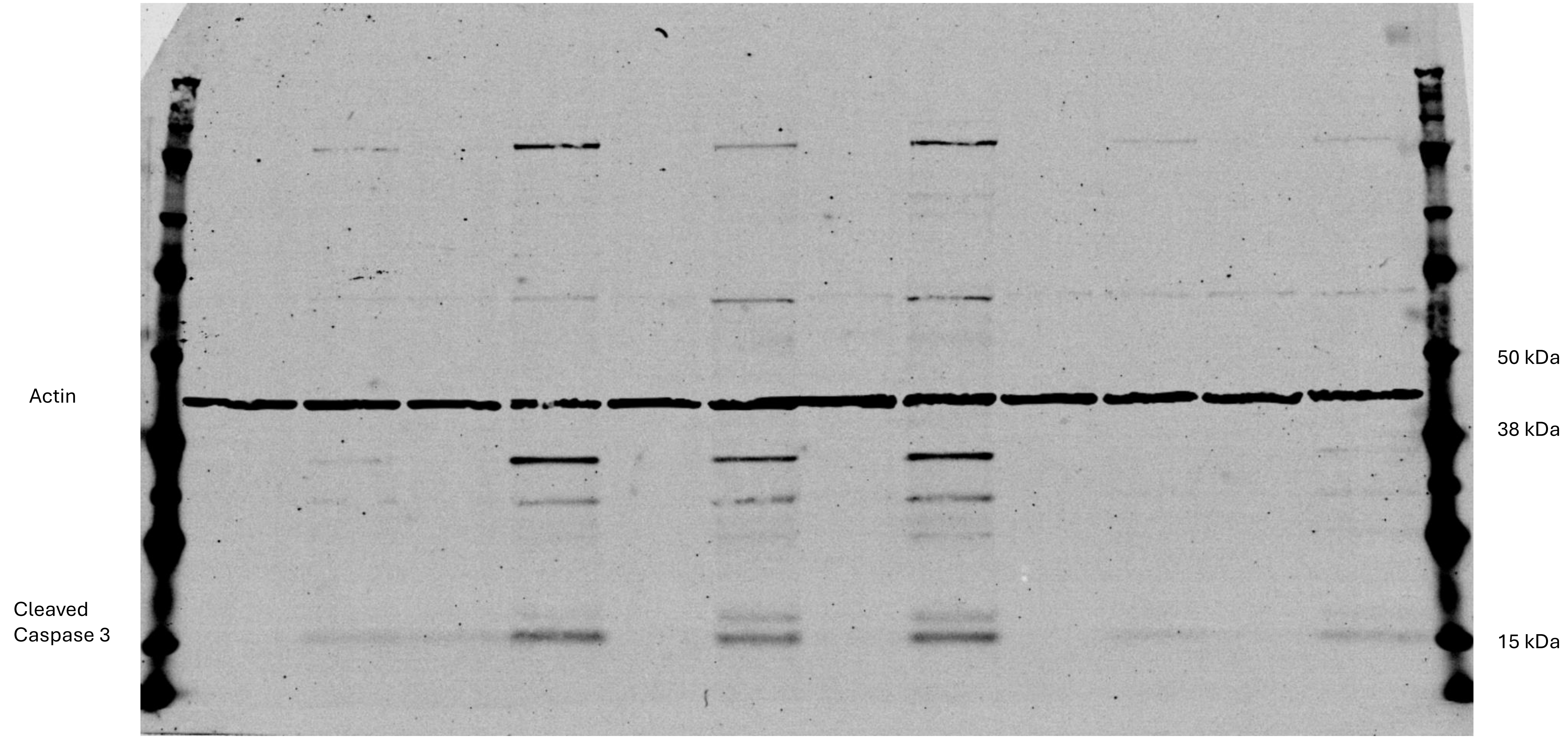

CAL51, SCR, P53-10

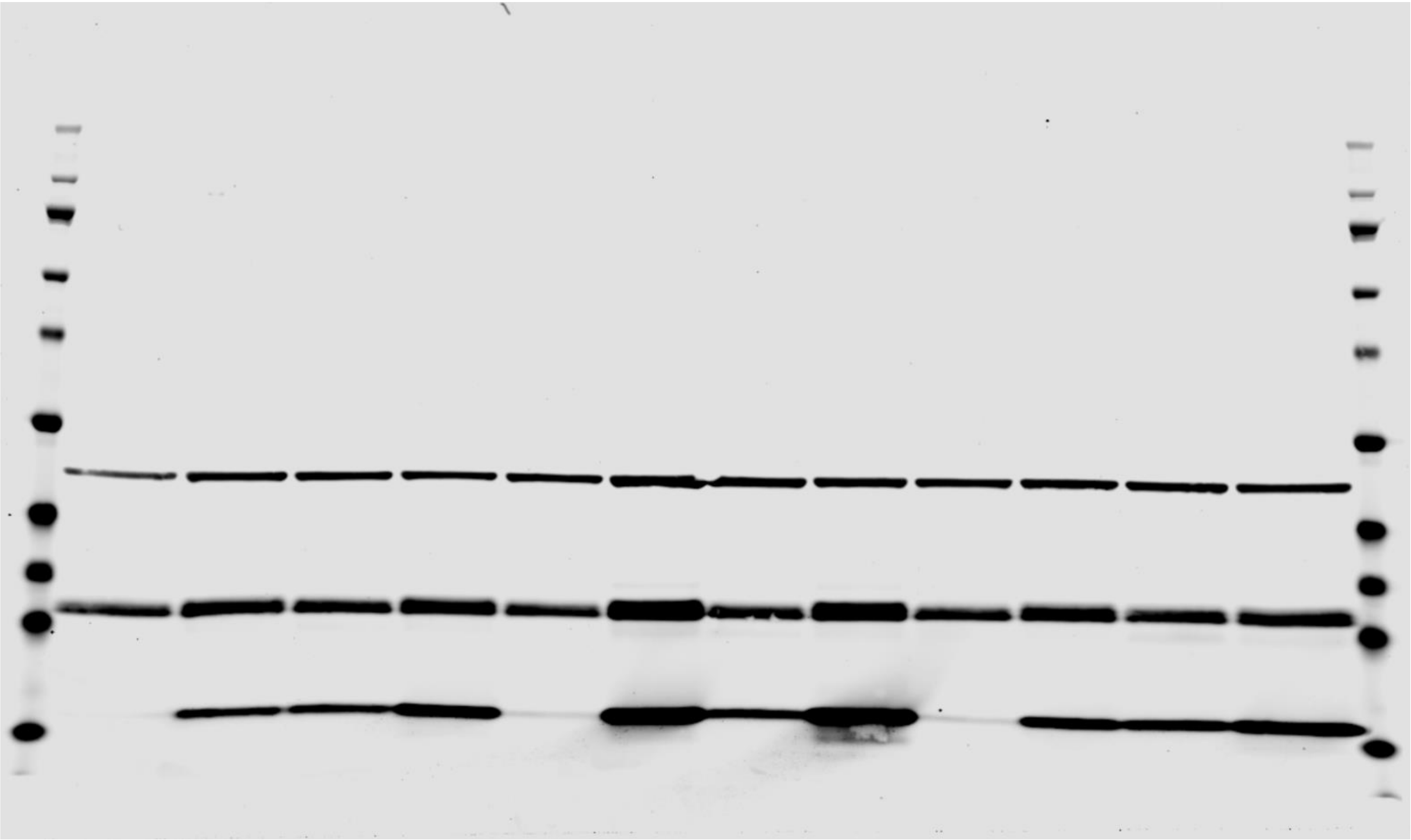

CAL51, SCR, P53-10

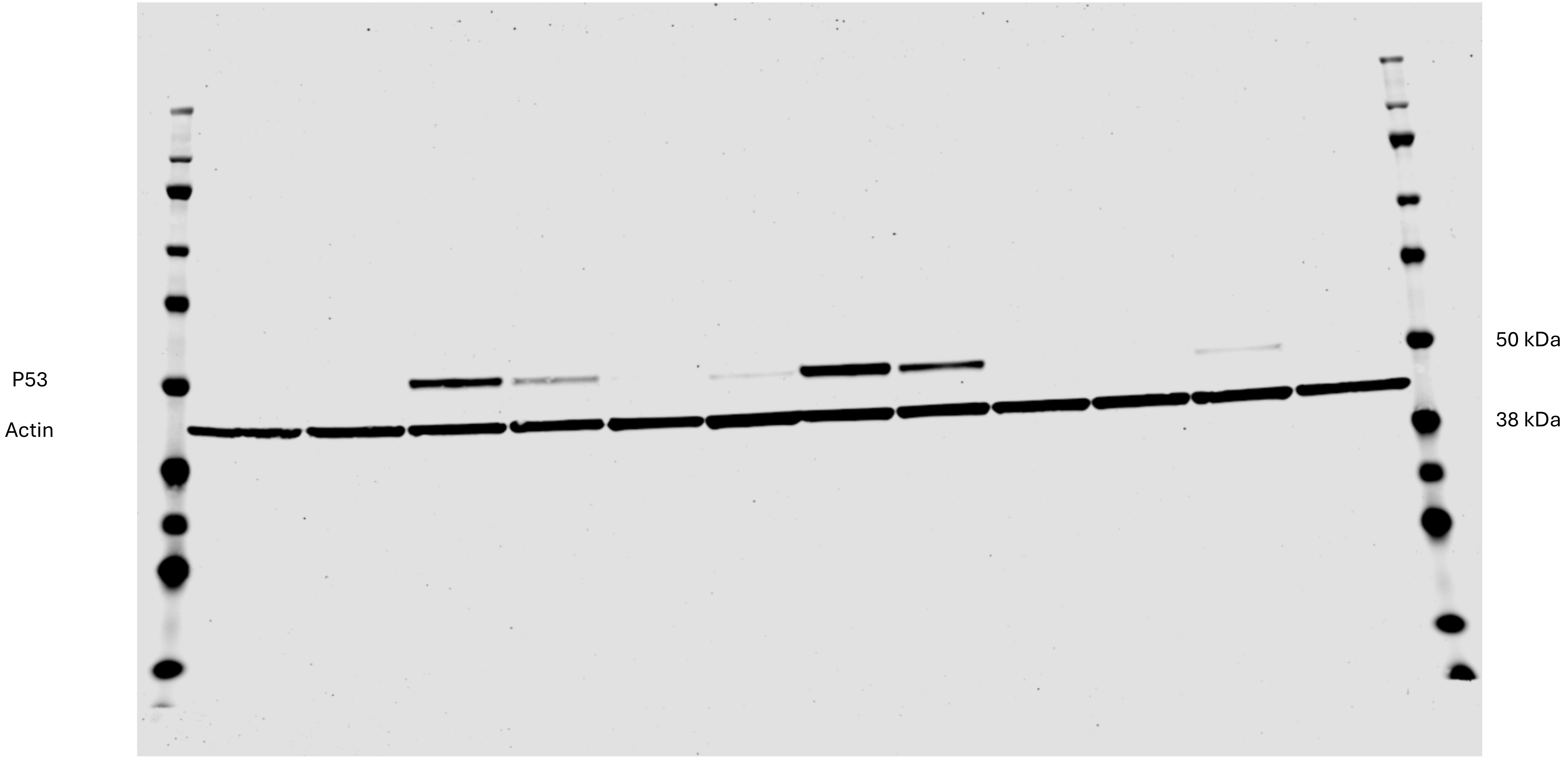

CAL51, SCR, P53-10

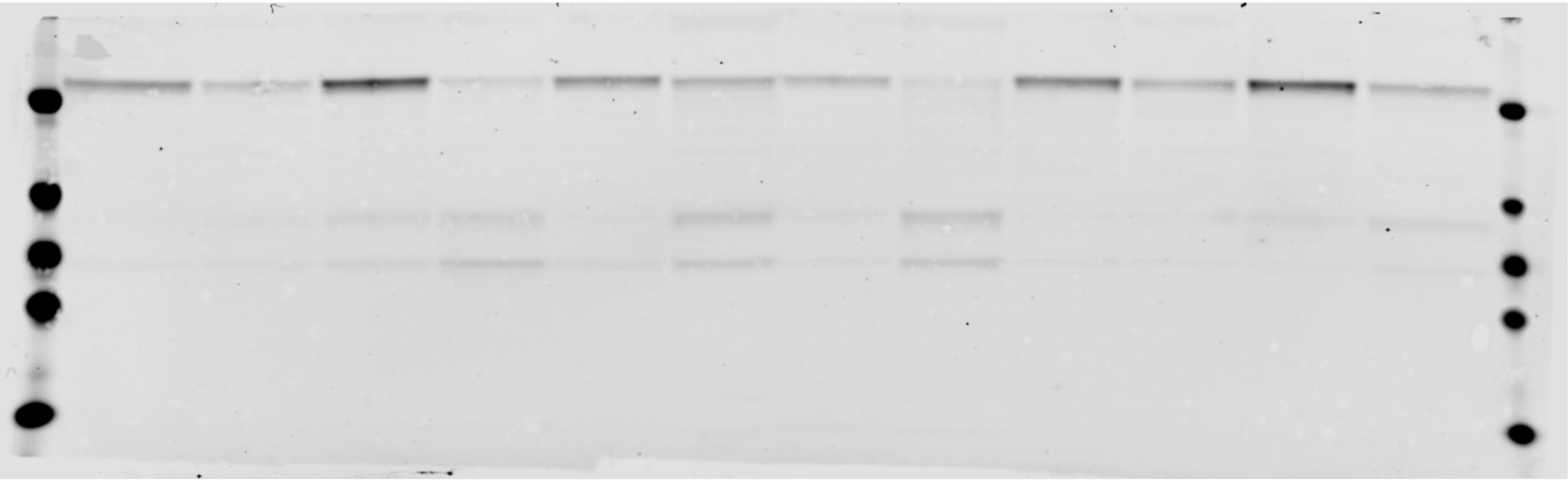

CAL51, SCR, P53-10

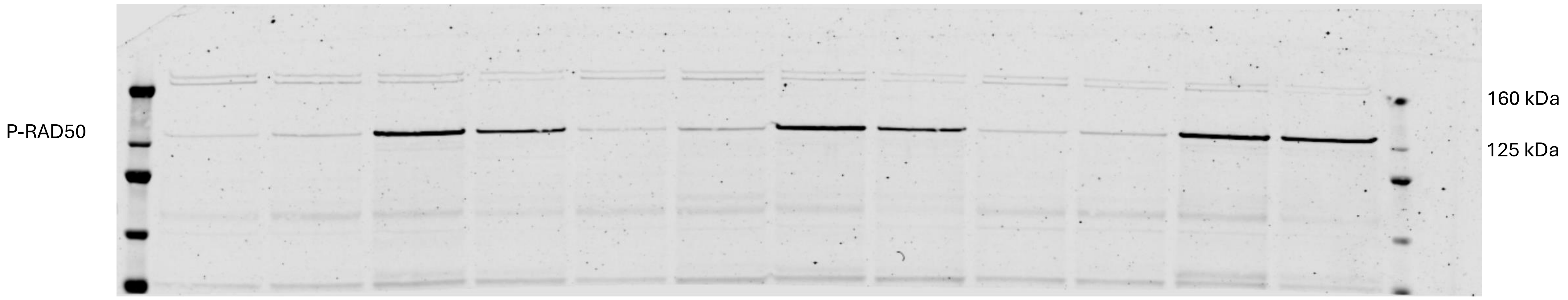

CAL51, SCR, P53-10

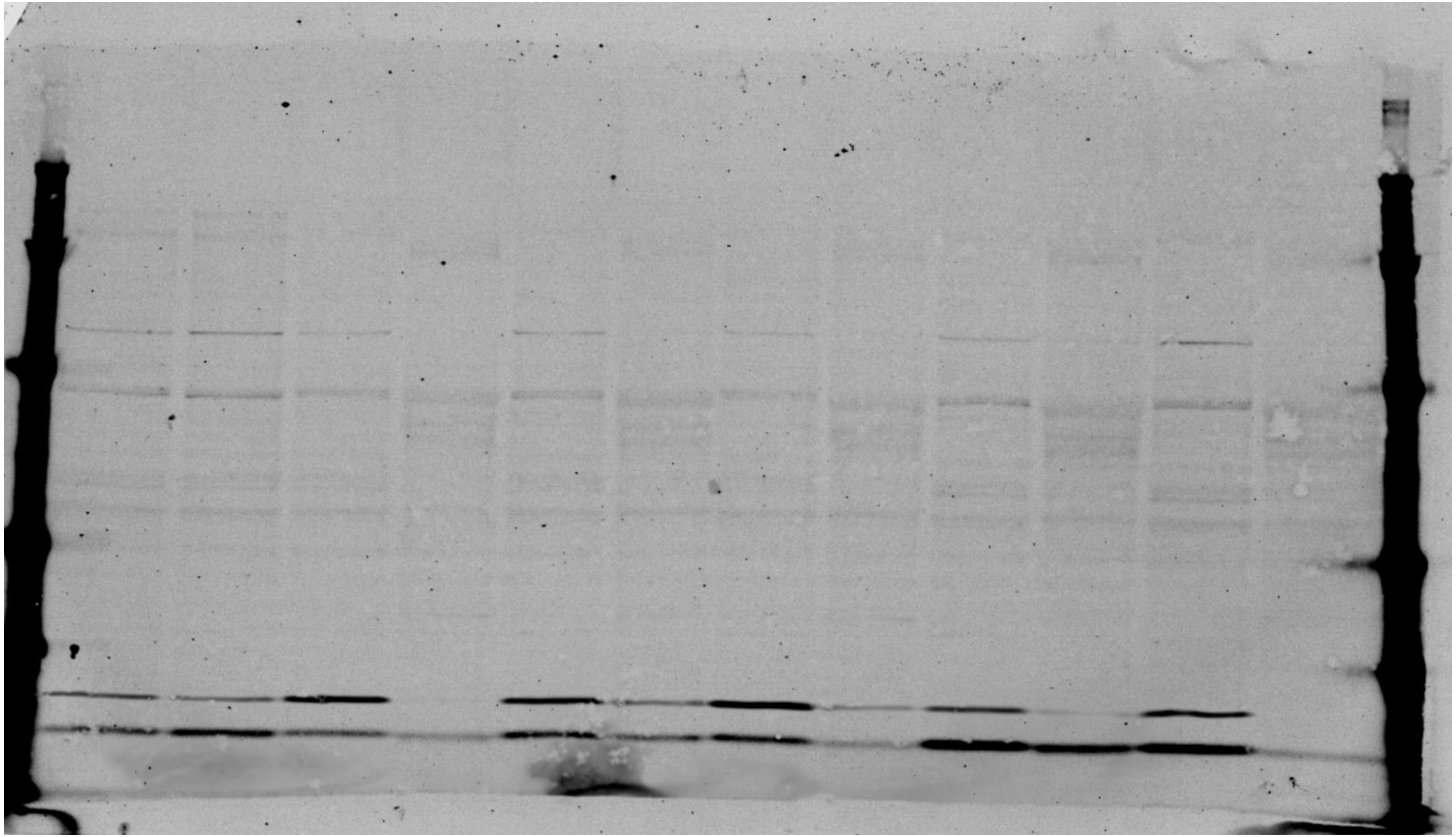

P16

25 kDa

15 kDa

CAL51, SCR, P53-10

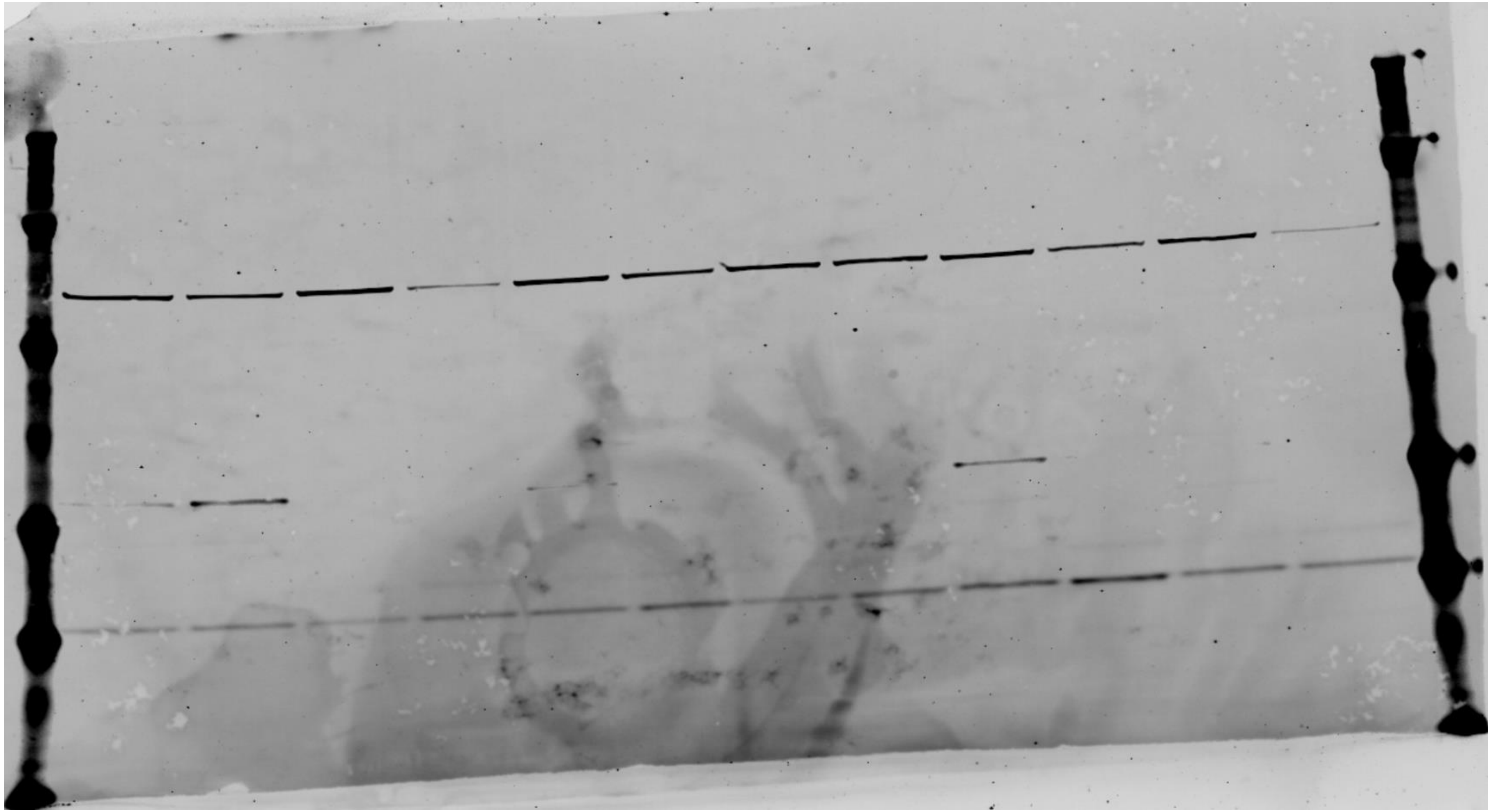

25 kDa

15 kDa

P21

CAL51, SCR, P53-10

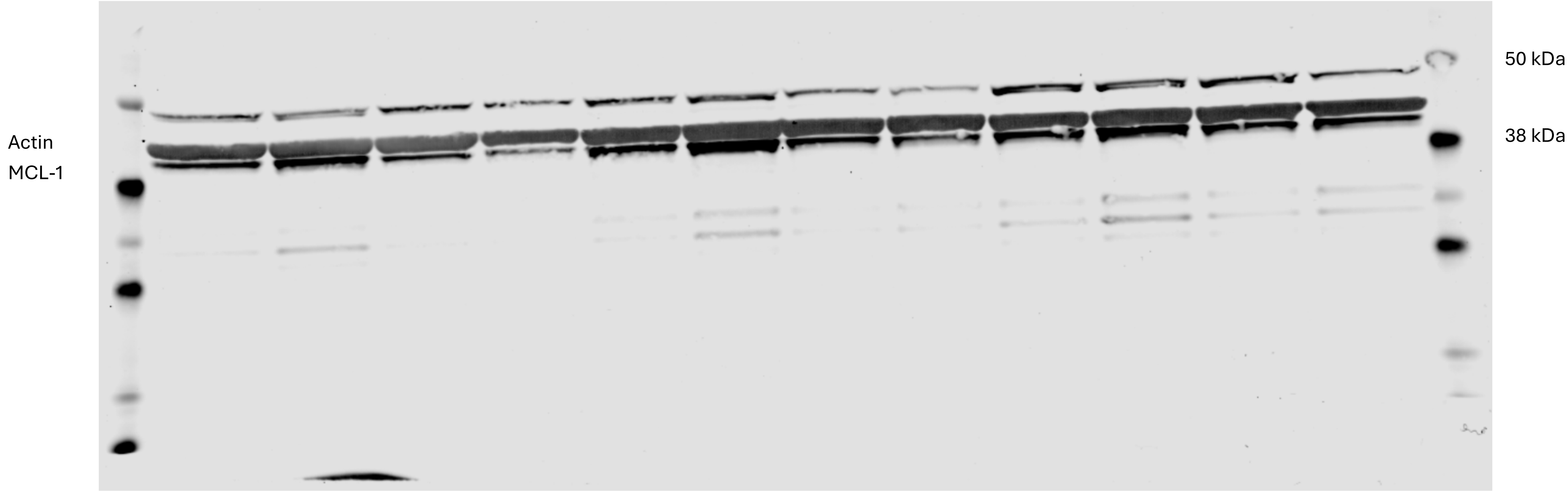

CAL51, SCR, P53-10

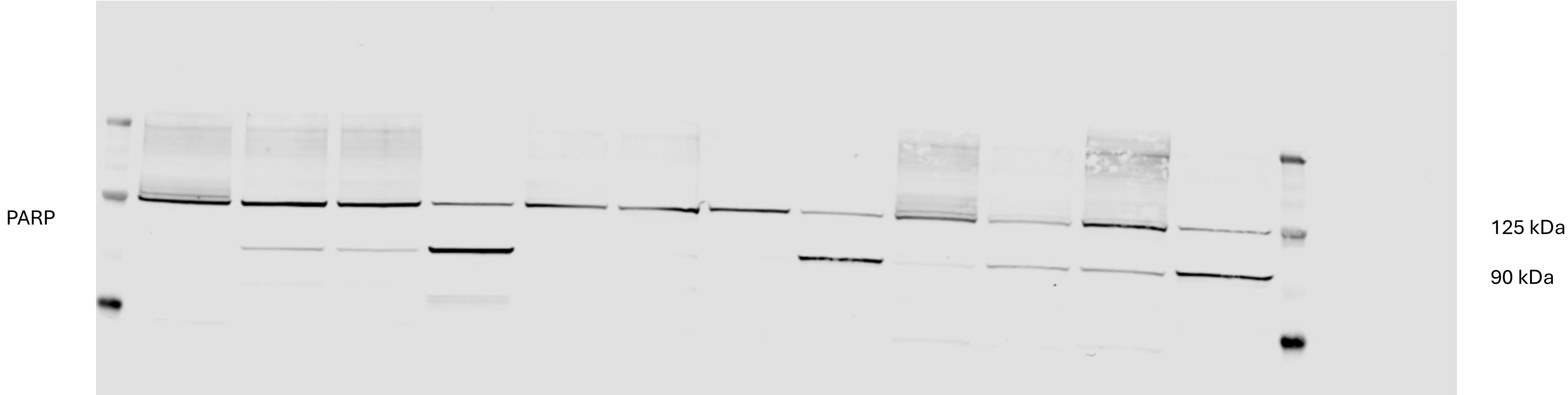

Supplement: Supplementary file 1 [file DataSheet1.pdf]
