# Supplementary material for: Potentiating doxorubicin activity through BCL-2 inhibition in p53 wild-type and mutated triple-negative breast cancer
Source: Front Oncol. 2025 Apr 2;15:1549282. doi: 10.3389/fonc.2025.1549282 (PMC11999952; doi:10.3389/fonc.2025.1549282)
Supplement: Supplementary file 2 [file Table1.docx]

|  |  | P-Value |  |  | P-value |  |  | P-Value |
| --- | --- | --- | --- | --- | --- | --- | --- | --- |
|  |  |  |  |  |  |  |  |  |
| CAL-51 | G1 | |  | S | |  | G2M | |
| ND vs. DOX | ** | 0.0035 |  | * | 0.0292 |  | ** | 0.003 |
| ND vs. VEN | *** | 0.0005 |  | ns | 0.0607 |  | ns | 0.4809 |
| ND vs. DOX/VEN | ns | 0.9691 |  | ns | 0.5087 |  | ns | 0.8535 |
| DOX vs. VEN | **** | <0.0001 |  | ns | 0.9512 |  | *** | 0.0006 |
| DOX vs. DOX/VEN | ** | 0.006 |  | ** | 0.0044 |  | ** | 0.0012 |
| VEN  vs. DOX/VEN | *** | 0.0004 |  | ** | 0.0085 |  | ns | 0.8942 |
|  |  |  |  |  |  |  |  |  |
| MDA-MB-231 | G1 | |  | S | |  | G2M | |
| ND vs. DOX | **** | <0.0001 |  | ns | 0.3151 |  | ** | 0.004 |
| ND vs. VEN | *** | 0.0005 |  | ns | 0.2354 |  | ns | 0.8248 |
| ND vs. DOX/VEN | ** | 0.0031 |  | ns | 0.0823 |  | ns | 0.9924 |
| DOX vs. VEN | **** | <0.0001 |  | ns | 0.9955 |  | ** | 0.0015 |
| DOX vs. DOX/VEN | ** | 0.0092 |  | ** | 0.0066 |  | ** | 0.0029 |
| VEN  vs. DOX/VEN | **** | <0.0001 |  | ** | 0.005 |  | ns | 0.9329 |
|  |  |  |  |  |  |  |  |  |
| Hs578t | G1 | |  | S | |  | G2M | |
| ND vs. DOX | *** | 0.0005 |  | * | 0.011 |  | ns | 0.5327 |
| ND vs. VEN | ** | 0.0056 |  | ns | 0.2263 |  | ns | 0.9316 |
| ND vs. DOX/VEN | * | 0.0327 |  | ns | 0.9592 |  | ns | 0.0626 |
| DOX vs. VEN | **** | <0.0001 |  | *** | 0.0009 |  | ns | 0.849 |
| DOX vs. DOX/VEN | * | 0.0344 |  | ** | 0.0059 |  | ** | 0.0093 |
| VEN  vs. DOX/VEN | *** | 0.0001 |  | ns | 0.4164 |  | * | 0.0273 |
|  |  |  |  |  |  |  |  |  |
| CAL120 | G1 | |  | S | |  | G2M | |
| ND vs. DOX | **** | <0.0001 |  | ns | 0.8813 |  | ** | 0.005 |
| ND vs. VEN | **** | <0.0001 |  | ns | 0.1302 |  | ns | 0.7059 |
| ND vs. DOX/VEN | * | 0.0254 |  | ns | 0.9207 |  | ns | 0.934 |
| DOX vs. VEN | **** | <0.0001 |  | ns | 0.3439 |  | ** | 0.0014 |
| DOX vs. DOX/VEN | *** | 0.0001 |  | ns | 0.5551 |  | * | 0.0104 |
| VEN  vs. DOX/VEN | **** | <0.0001 |  | ns | 0.0539 |  | ns | 0.3956 |
|  |  |  |  |  |  |  |  |  |

**Supplemental Table 1**: Statistical analysis of the cell cycle (Figure 3C). Analysis performed using one-way ANOVA with Tukey correction.
