# Supplementary material for: Potentiating doxorubicin activity through BCL-2 inhibition in p53 wild-type and mutated triple-negative breast cancer
Source: Front Oncol. 2025 Apr 2;15:1549282. doi: 10.3389/fonc.2025.1549282 (PMC11999952; doi:10.3389/fonc.2025.1549282)
Supplement: Supplementary file 3 [file Presentation1.pptx]

## Slide 1
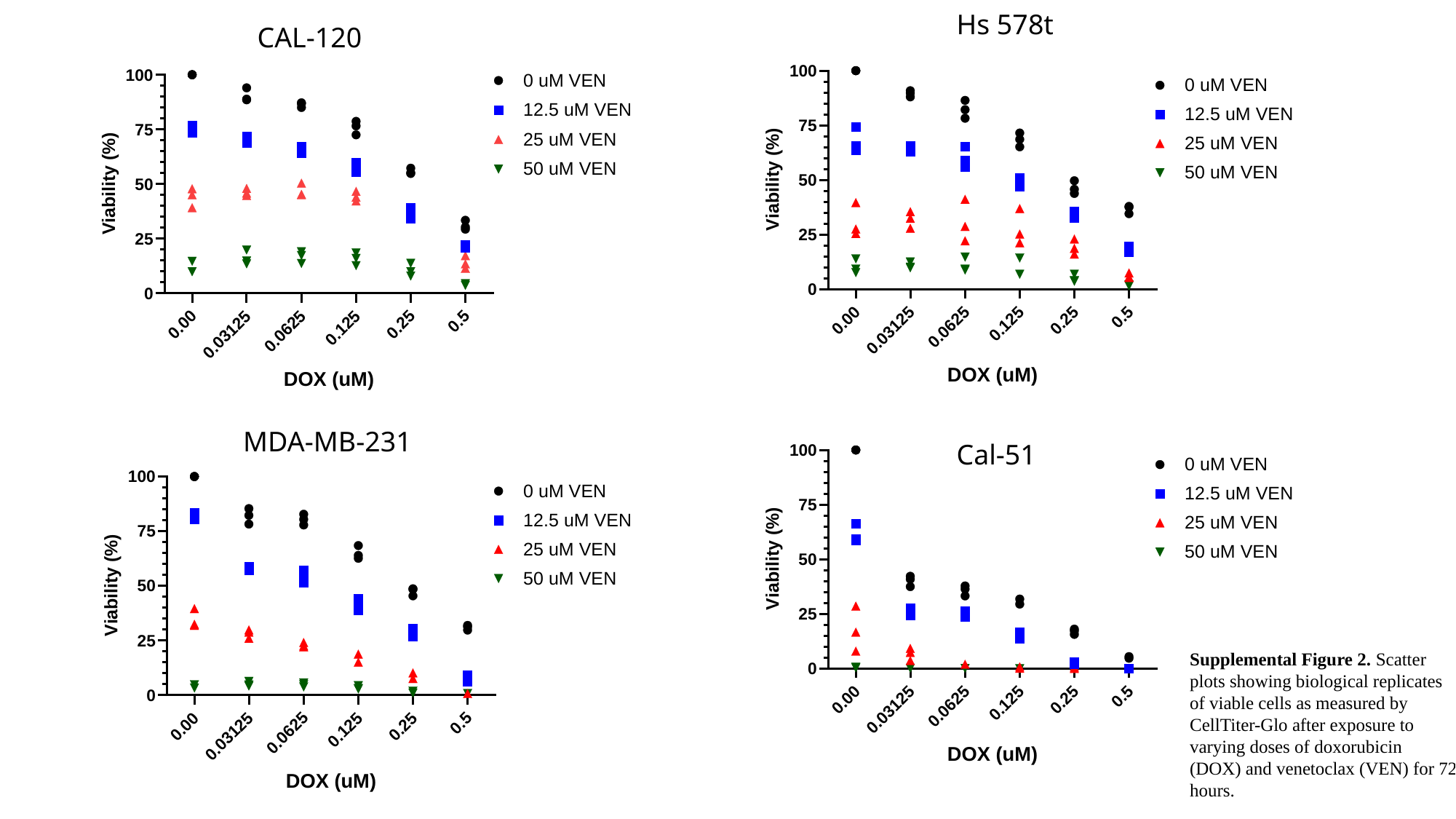

Hs 578t
CAL-120
MDA-MB-231
Cal-51
Supplemental Figure 2. Scatter plots showing biological replicates of viable cells as measured by CellTiter-Glo after exposure to varying doses of doxorubicin (DOX) and venetoclax (VEN) for 72 hours.

## Slide 2
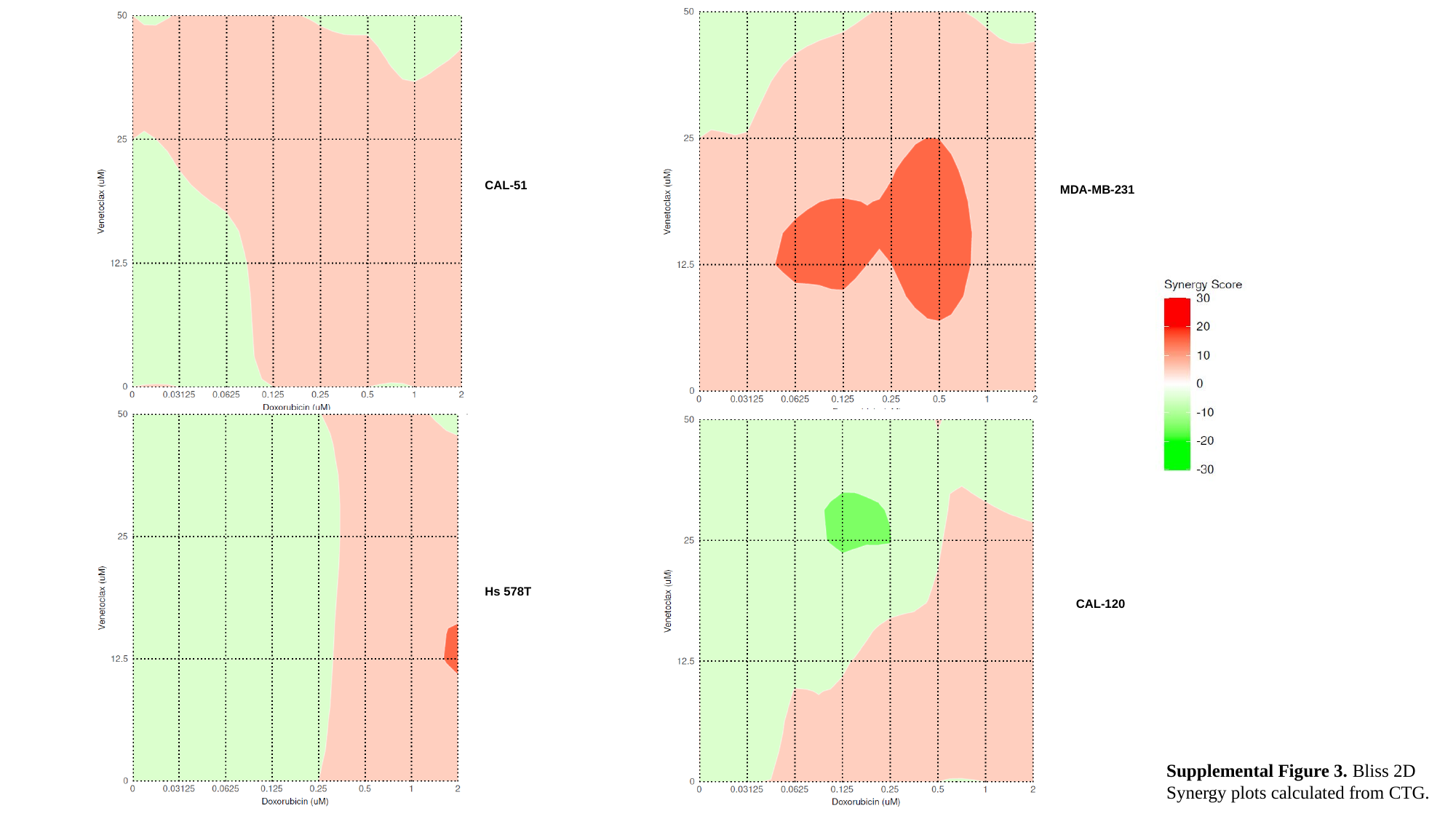

CAL-51
MDA-MB-231
Hs 578T
CAL-120
Supplemental Figure 3. Bliss 2D Synergy plots calculated from CTG.

## Slide 3
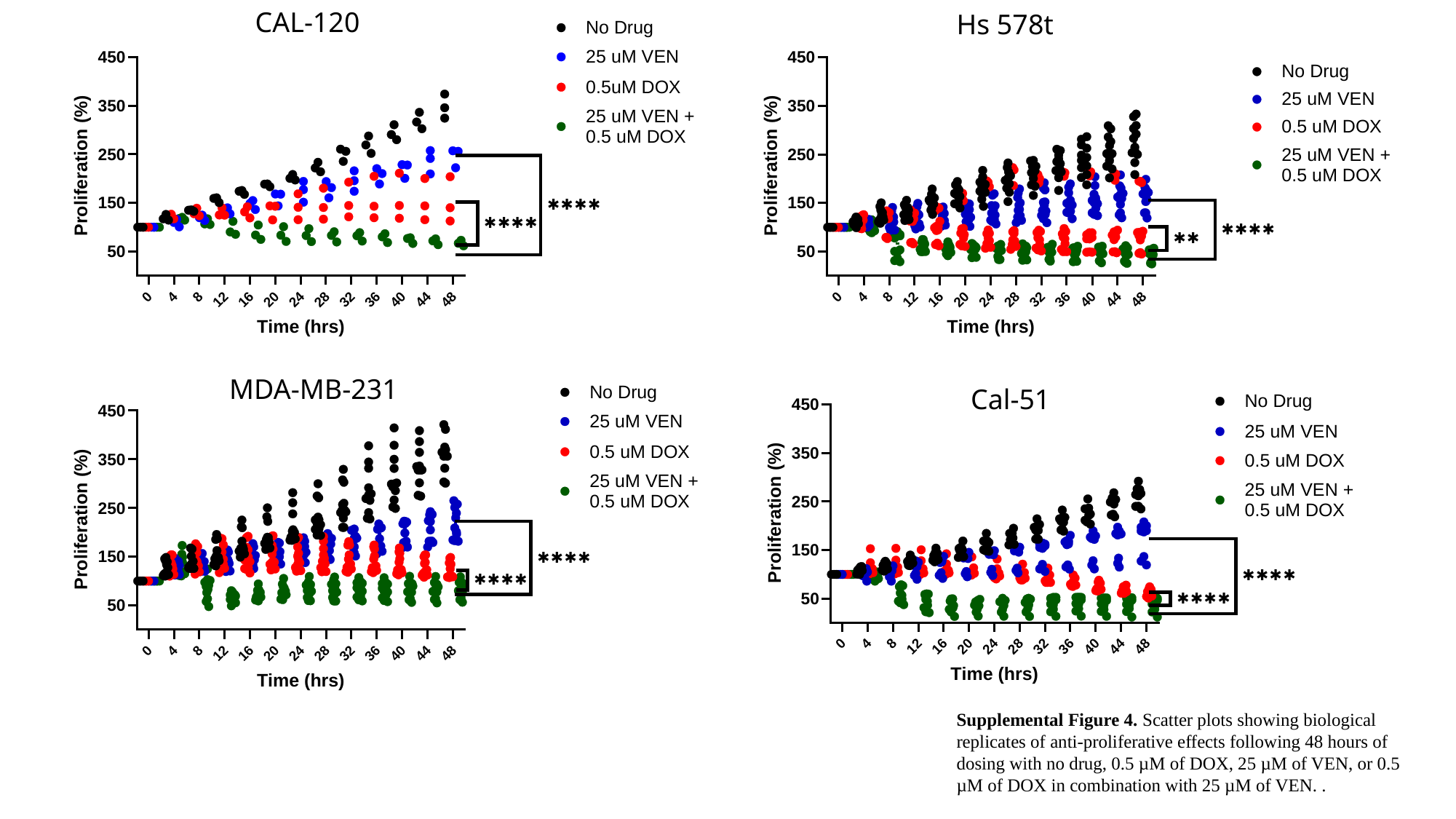

CAL-120
Hs 578t
MDA-MB-231
Cal-51
Supplemental Figure 4. Scatter plots showing biological replicates of anti-proliferative effects following 48 hours of dosing with no drug, 0.5 µM of DOX, 25 µM of VEN, or 0.5 µM of DOX in combination with 25 µM of VEN. .

## Slide 4
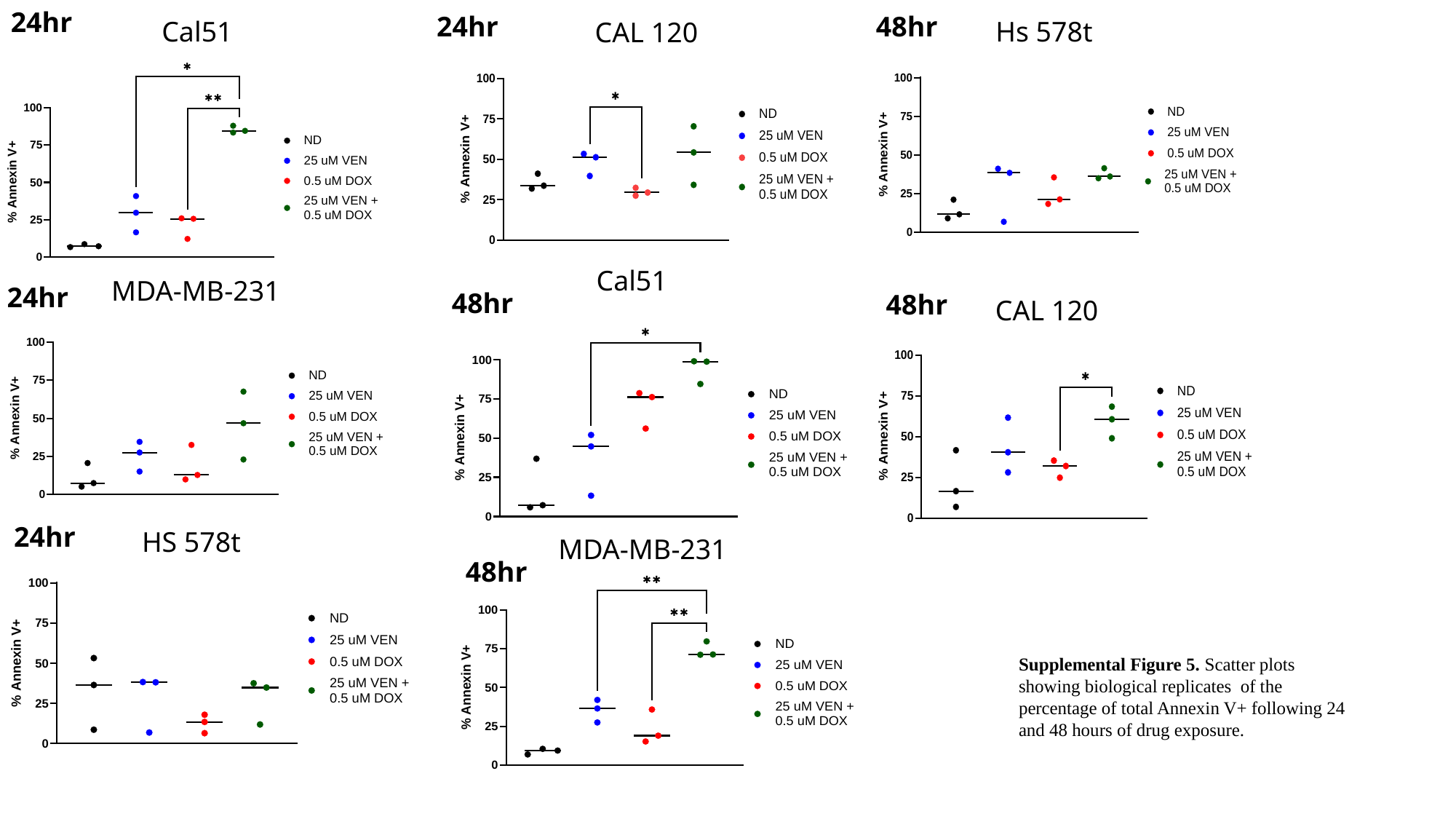

24hr
24hr
48hr
Cal51
Hs 578t
CAL 120
Cal51
MDA-MB-231
24hr
48hr
48hr
CAL 120
24hr
HS 578t
MDA-MB-231
48hr
Supplemental Figure 5. Scatter plots showing biological replicates of the percentage of total Annexin V+ following 24 and 48 hours of drug exposure.

## Slide 5
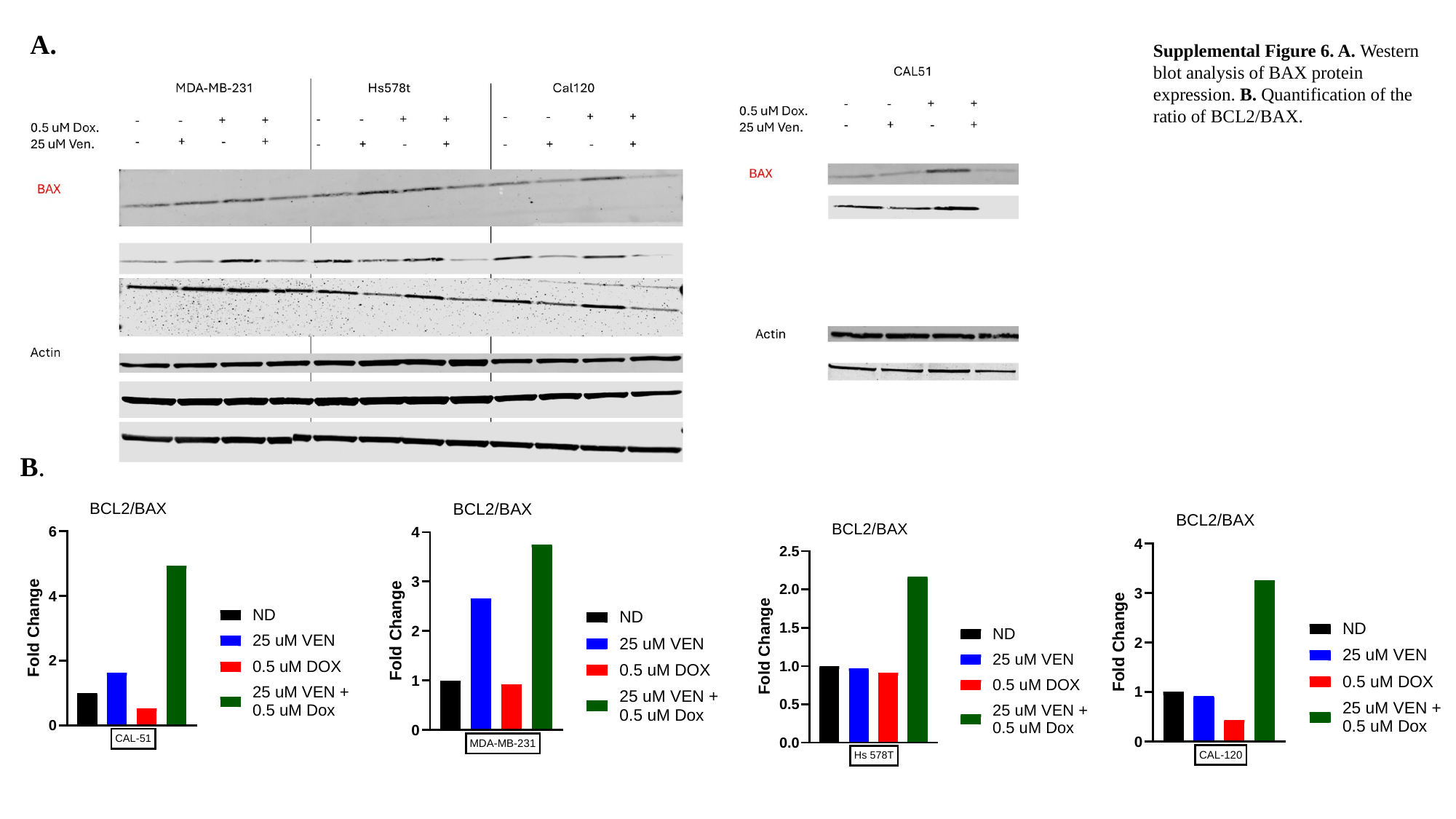

A.
Supplemental Figure 6. A. Western blot analysis of BAX protein expression. B. Quantification of the ratio of BCL2/BAX.
B.

## Slide 6
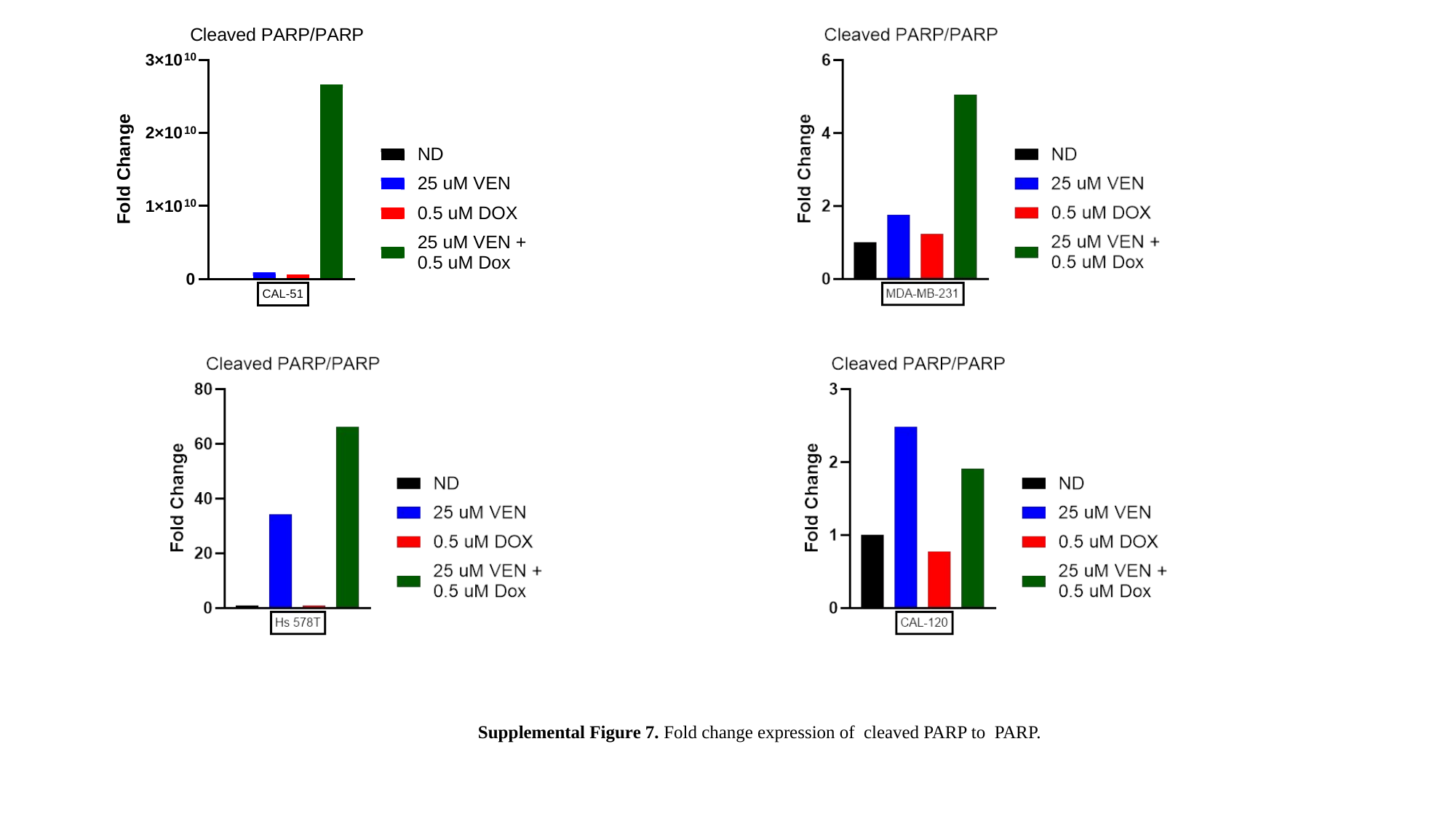

Supplemental Figure 7. Fold change expression of cleaved PARP to PARP.

## Slide 7
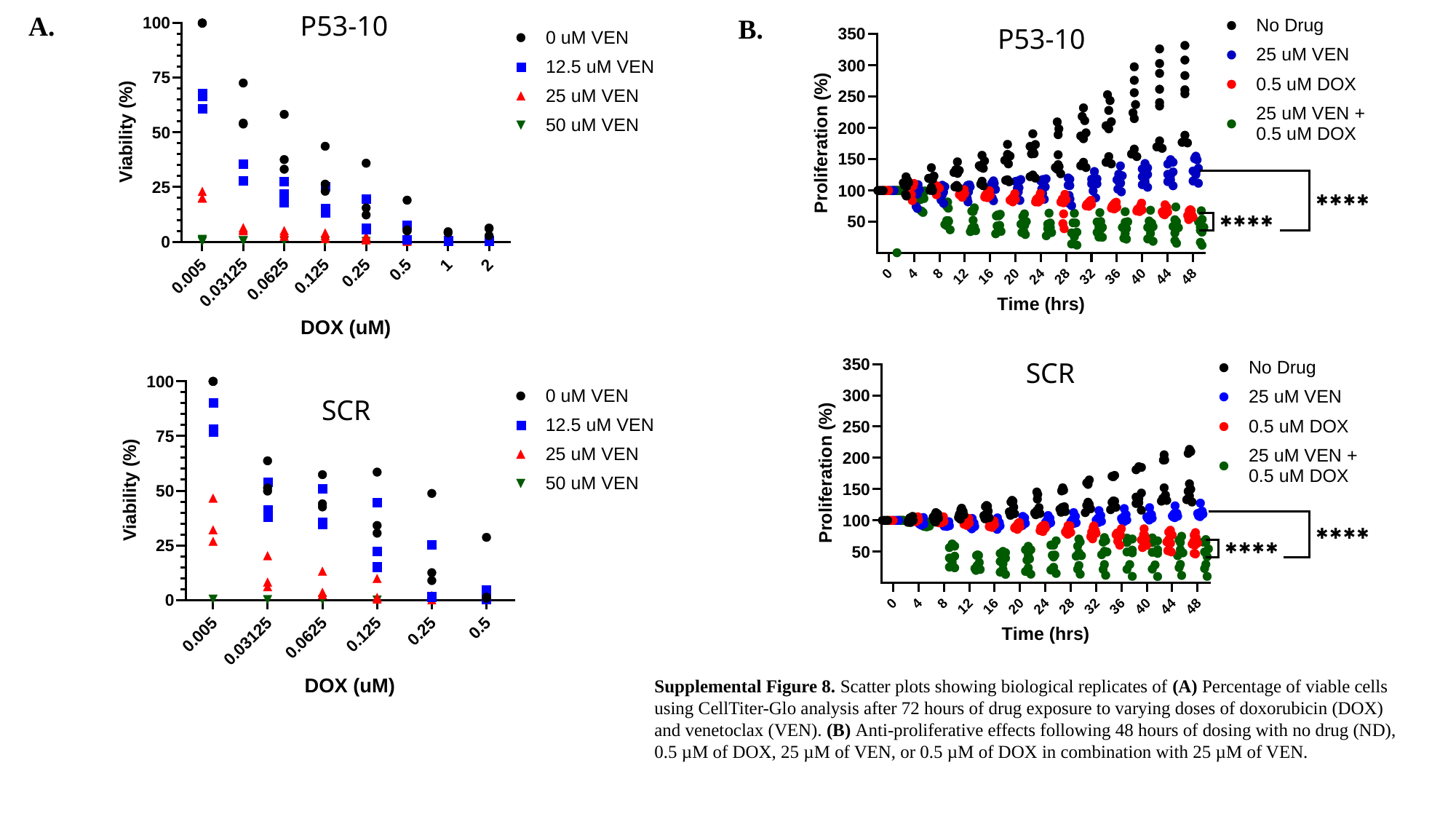

A.
P53-10
B.
P53-10
SCR
SCR
Supplemental Figure 8. Scatter plots showing biological replicates of (A) Percentage of viable cells using CellTiter-Glo analysis after 72 hours of drug exposure to varying doses of doxorubicin (DOX) and venetoclax (VEN). (B) Anti-proliferative effects following 48 hours of dosing with no drug (ND), 0.5 µM of DOX, 25 µM of VEN, or 0.5 µM of DOX in combination with 25 µM of VEN.

## Slide 8
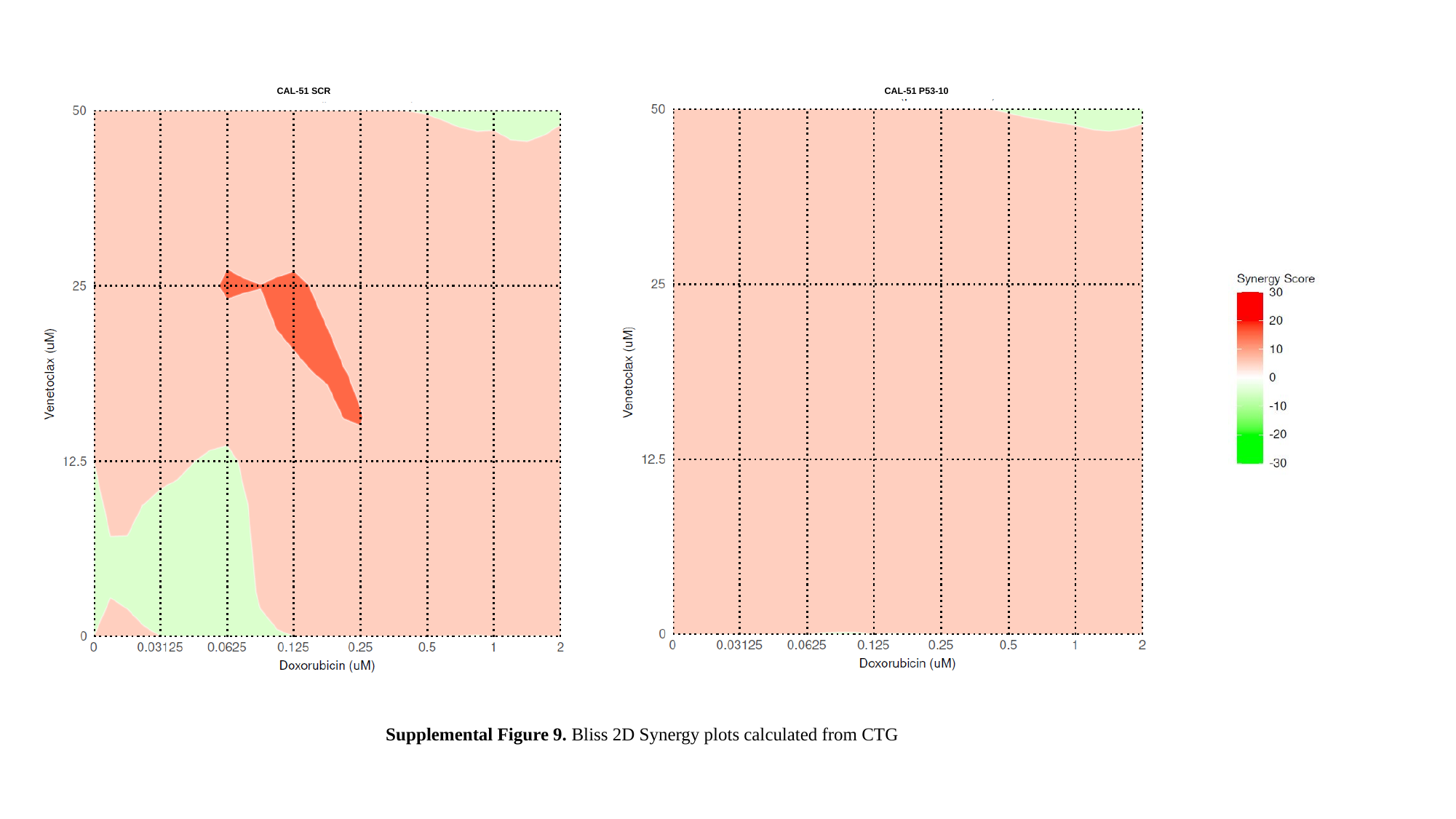

CAL-51 SCR
CAL-51 P53-10
Supplemental Figure 9. Bliss 2D Synergy plots calculated from CTG

## Slide 9
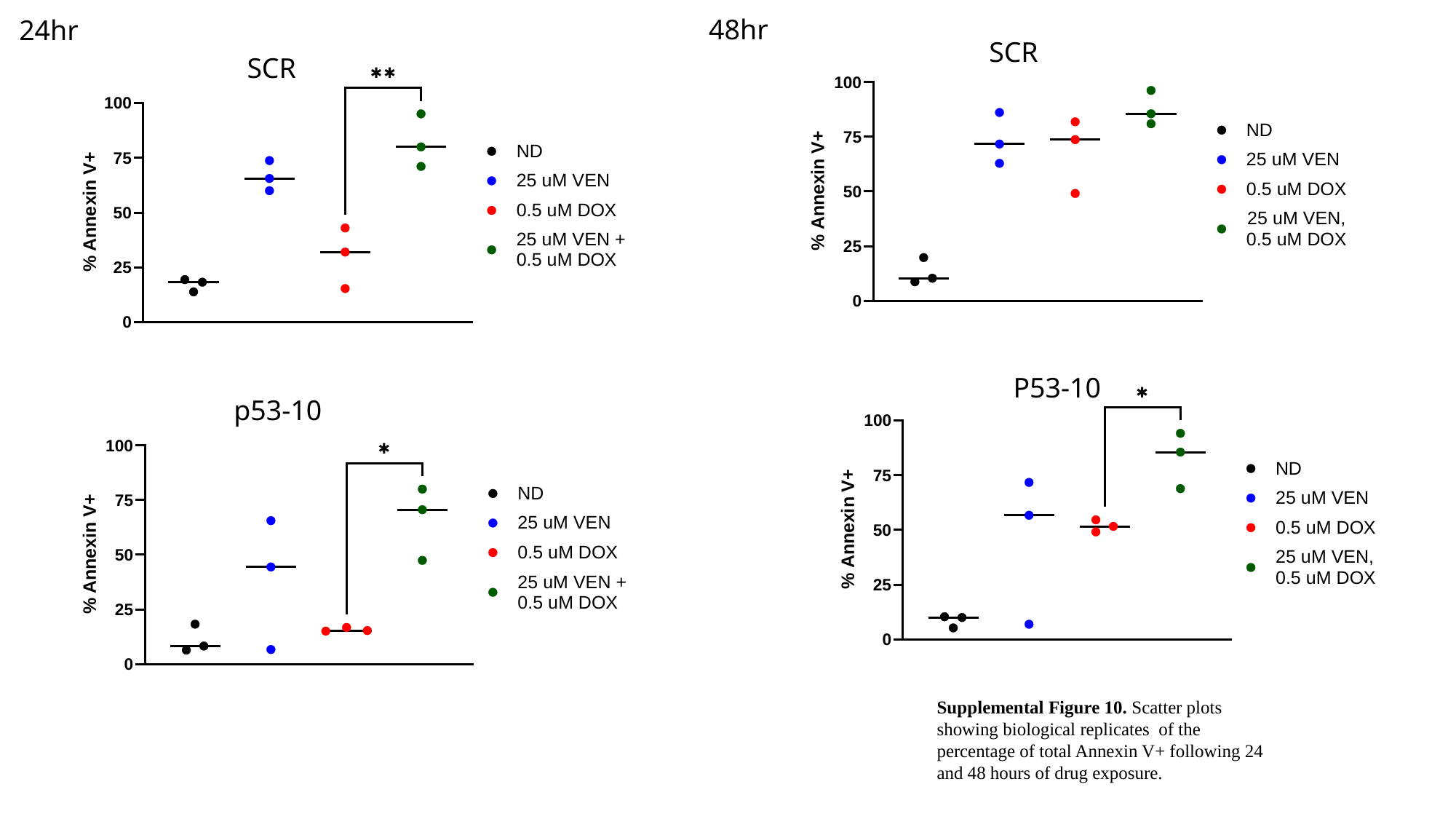

48hr
24hr
SCR
SCR
P53-10
p53-10
Supplemental Figure 10. Scatter plots showing biological replicates of the percentage of total Annexin V+ following 24 and 48 hours of drug exposure.

## Slide 10
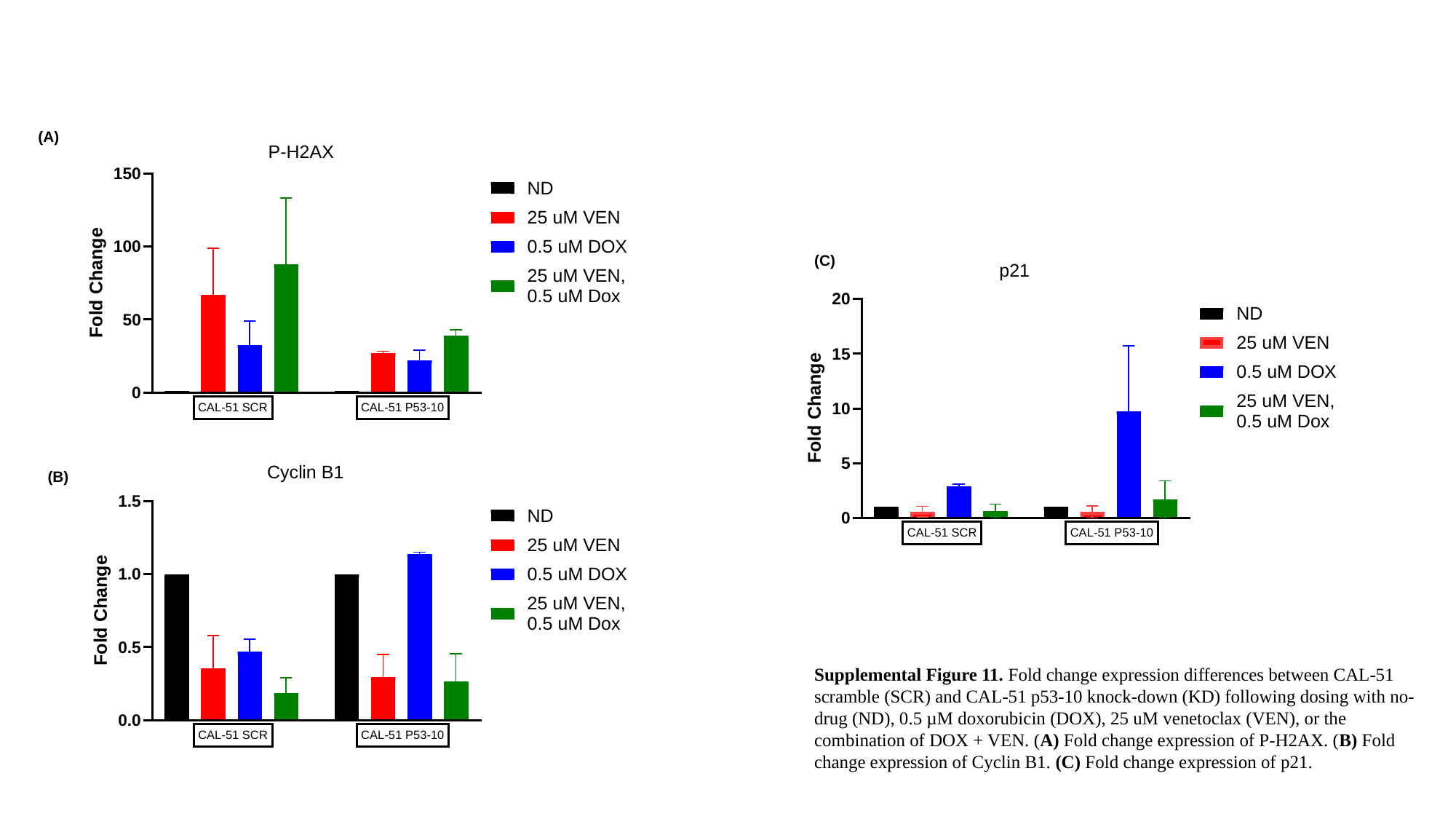

(A)
P-H2AX
(C)
(B)
Supplemental Figure 11. Fold change expression differences between CAL-51 scramble (SCR) and CAL-51 p53-10 knock-down (KD) following dosing with no-drug (ND), 0.5 µM doxorubicin (DOX), 25 uM venetoclax (VEN), or the combination of DOX + VEN. (A) Fold change expression of P-H2AX. (B) Fold change expression of Cyclin B1. (C) Fold change expression of p21.

## Slide 11
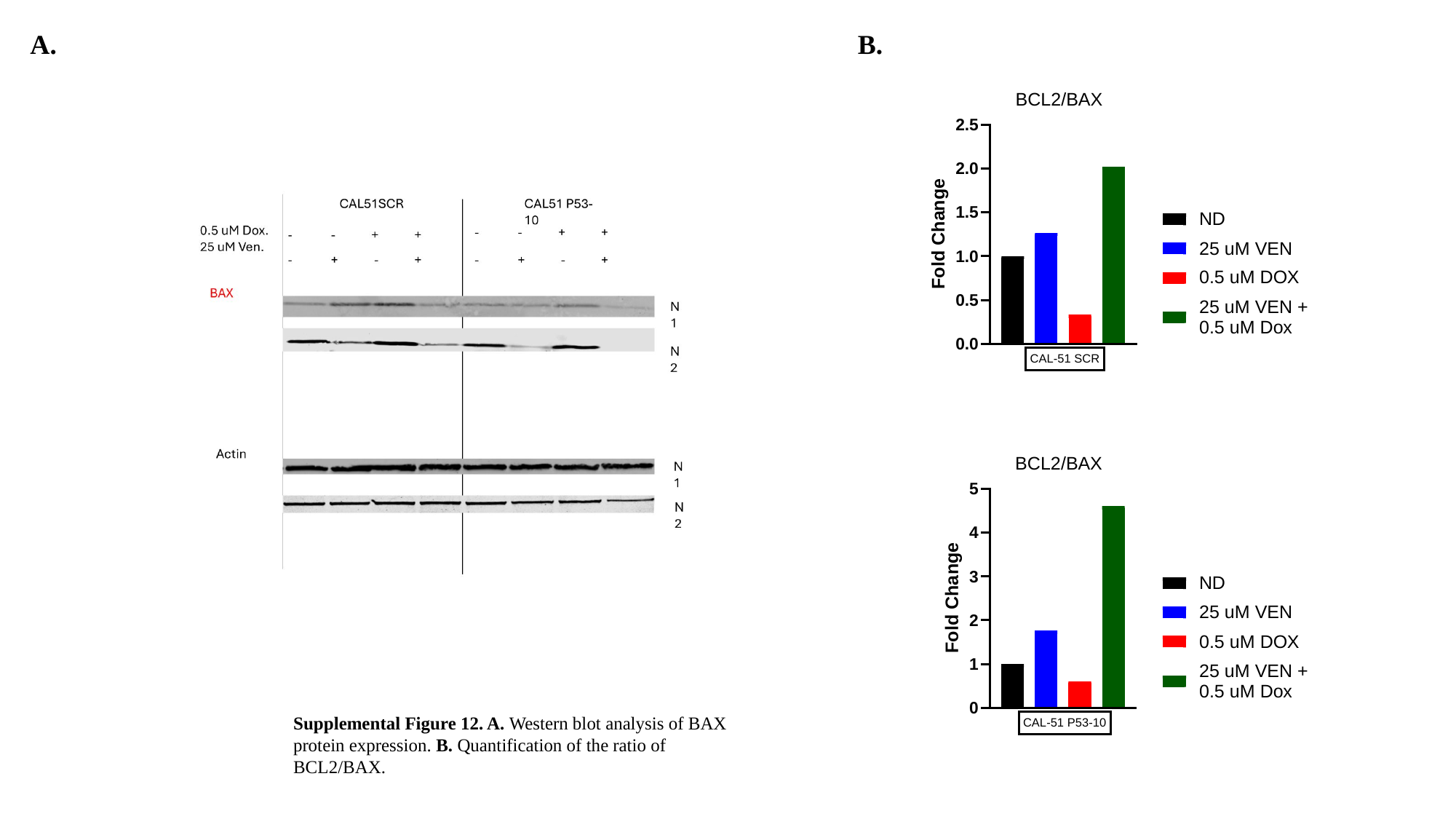

A.
B.
Supplemental Figure 12. A. Western blot analysis of BAX protein expression. B. Quantification of the ratio of BCL2/BAX.

## Slide 12
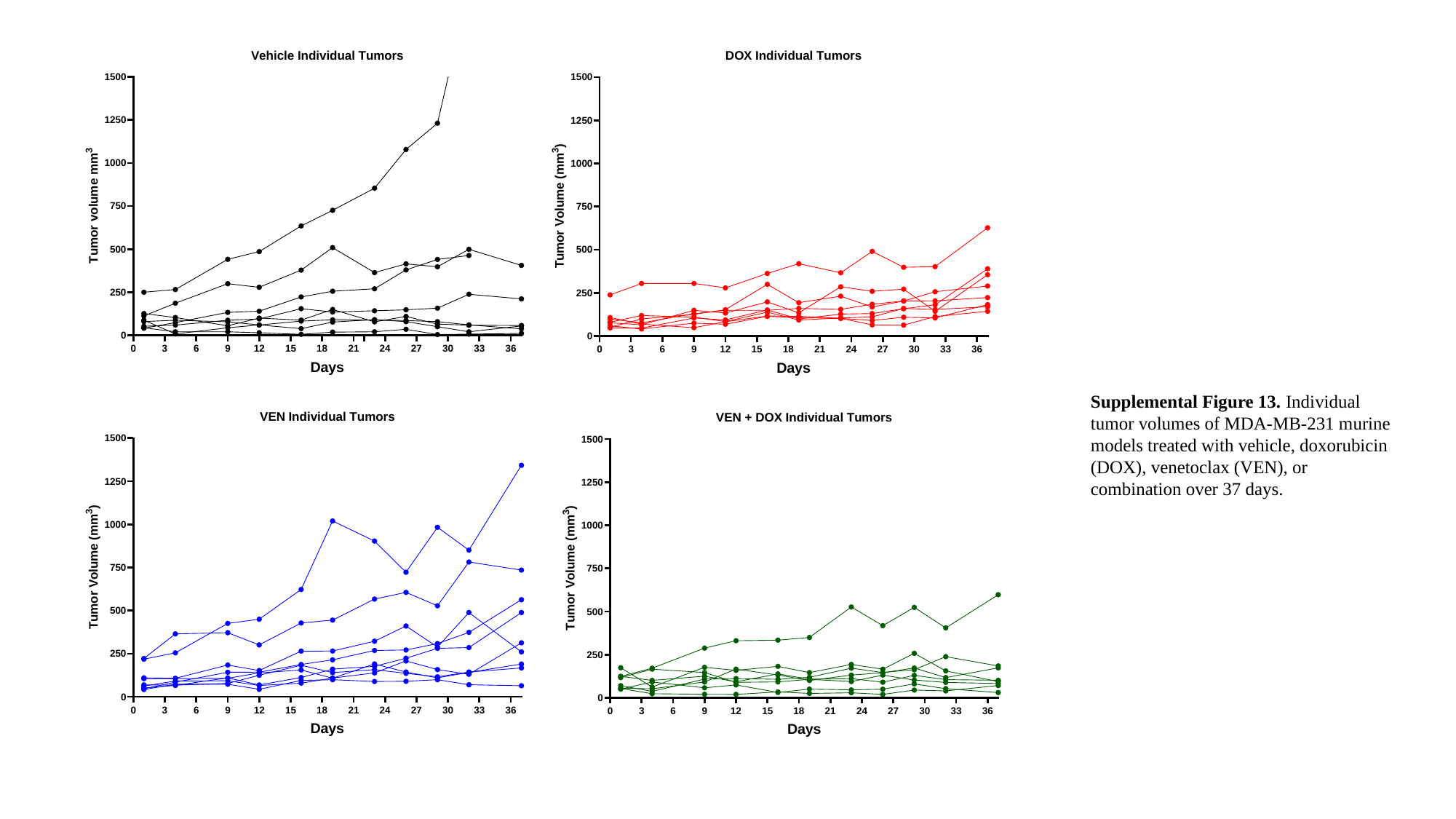

Supplemental Figure 13. Individual tumor volumes of MDA-MB-231 murine models treated with vehicle, doxorubicin (DOX), venetoclax (VEN), or combination over 37 days.

## Slide 13
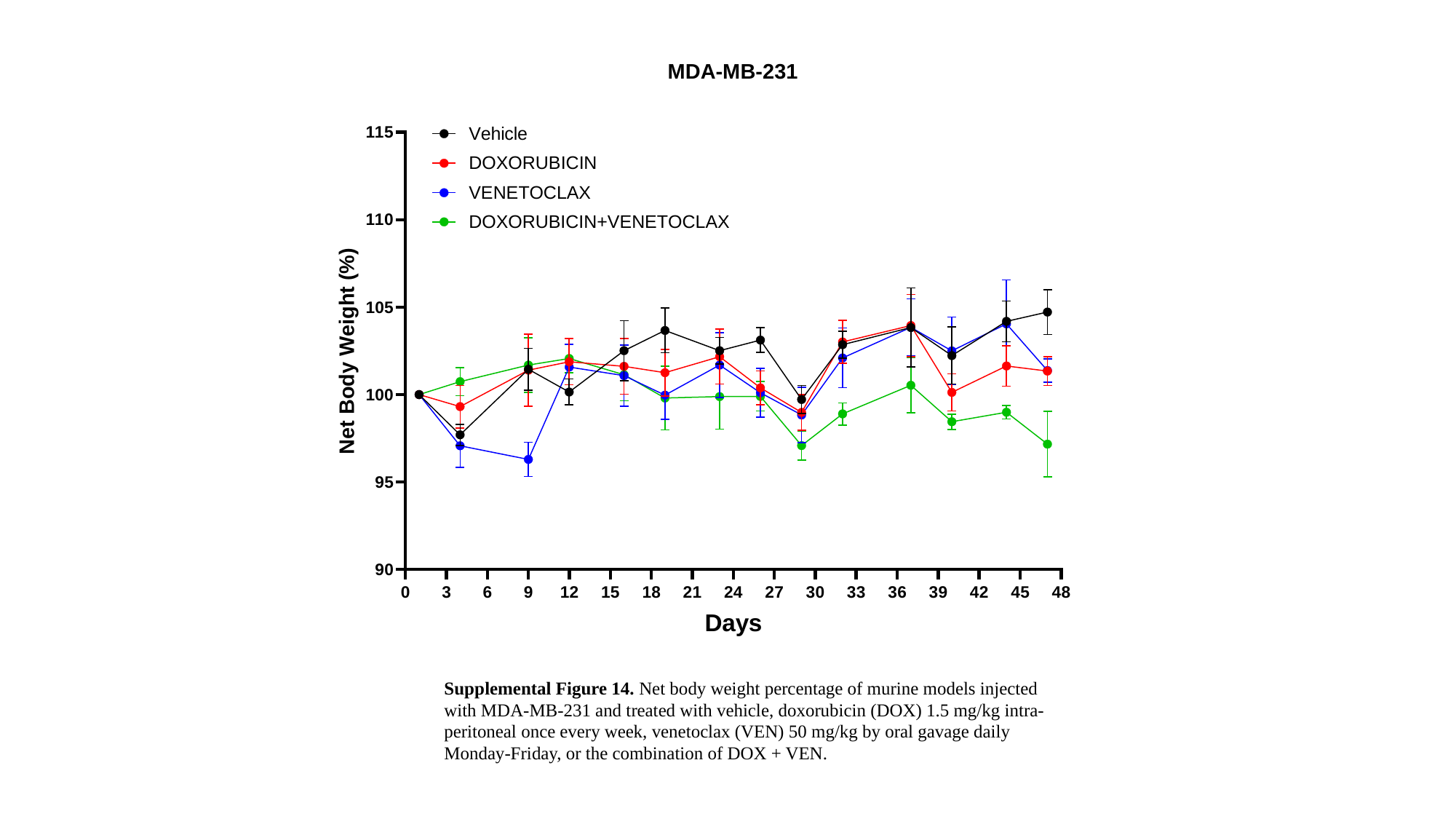

Supplemental Figure 14. Net body weight percentage of murine models injected with MDA-MB-231 and treated with vehicle, doxorubicin (DOX) 1.5 mg/kg intra-peritoneal once every week, venetoclax (VEN) 50 mg/kg by oral gavage daily Monday-Friday, or the combination of DOX + VEN.
